# Operando Electrochemical Formation of Integrated Ni‐Fe Oxyhydroxide Anode for Durable Anion Exchange Membrane Water Electrolyzer

**DOI:** 10.1002/advs.202600055

**Published:** 2026-06-22

**Authors:** Euntaek Oh, Jonghyun Hyun, Hojin Lee, Changsoo Lee, Jang Yong Lee, Dong Wook Lee, Kyunghwa Seok, Jeesoo Park, Susung Kim, Gisu Doo, Hee‐Tak Kim

**Affiliations:** ^1^ Department of Chemical and Biomolecular Engineering Korea Advanced Institute of Science and Technology (KAIST) Daejeon Republic of Korea; ^2^ Clean Energy Research Center Korea Institute of Science and Technology (KIST) Seoul Republic of Korea; ^3^ Department of Chemical Engineering Education Chungnam National University Daejeon Republic of Korea; ^4^ Department of Chemical Engineering Konkuk University Seoul Republic of Korea; ^5^ Hydrogen Research Department Korea Institute of Energy Research (KIER) Daejeon Republic of Korea

**Keywords:** anode, anion exchange membrane water electrolyzer, (Fe, Ni)OOH catalyst, integrated electrode, operando Fe doping

## Abstract

Efficient hydrogen production via anion exchange membrane water electrolyzers (AEMWEs) requires electrocatalysts that exhibit both high performance and long‐term stability under industrially relevant current densities. While Ni‐Fe‐based catalysts have emerged as promising candidates for the oxygen evolution reaction, their practical implementation in AEMWEs is still limited by multistep ex situ fabrication and, in many cases, by the poor interfacial stability of particle‐based catalyst layers. Here, we report an operando electrochemical strategy to directly construct integrated (Fe, Ni)OOH catalysts on Ni foam anodes via voltage‐cycling in a FeOOH‐containing electrolyte. This one‐step operando activation enables rapid and scalable fabrication of integrated electrodes in a practical electrolyzer device, eliminating the need for binders, slurry casting, and post‐annealing. The voltage cycling in the FeOOH‐containing electrolyte induces simultaneous Ni oxidation and Fe adsorption, leading to the formation of a porous, defect‐rich Ni‐O‐Fe structure with enhanced conductivity and catalytic activity. The resulting integrated electrode delivers a high current density of 2.8 A cm^−2^ at 1.8 V and operates stably for over 7000 h in a two‐cell AEMWE stack, without significant catalyst delamination. This work provides a simple, efficient, and scalable route for developing durable, non‐precious metal‐based anodes for AEMWEs.

## Graphical abstract

The interfacial stability and activity of Ni‐Fe‐based AEMWE anodes can be directly enhanced by operando voltage‐cycling in a FeOOH‐containing electrolyte. This process simultaneously induces Ni oxidation and Fe incorporation, forming an integrated, porous (Fe, Ni)OOH layer with high conductivity and durability. The operando‐formed electrode achieves high current densities and long‐term stability without relying on binders or multistep fabrication.

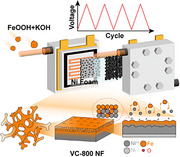

## 1 Introduction

Alkaline water electrolysis offers a promising approach to store surplus renewable energy in the form of chemical bonds, generating hydrogen and oxygen as clean fuels [1]. Despite extensive efforts to develop efficient electrocatalysts for the oxygen evolution reaction (OER; 4OH^−^ → O_2_ + 2H_2_O + 4e^−^), this anodic half‐reaction remains the primary bottleneck of overall water electrolysis, due to its complex four‐electron pathway [2]. To accelerate the OER kinetics, it is essential to develop electrocatalysts that manifest high activity, robust stability, and good electrical conductivity [3]. Noble‐metal‐based oxides, such as IrO_x_, have long been employed as reference OER catalysts because of their moderate catalytic activity in alkaline media [4]. However, their practical applicability is limited by poor chemical and mechanical stability of the electrodes (e.g., catalyst detachment and loss of electrical contact), as well as by their high cost and limited availability [5]. These limitations have prompted extensive research into alternative catalysts that are both economically viable and compatible with alkaline conditions.

Among various non‐noble metal catalysts, nickel–iron (Ni‐Fe)‐based materials have emerged as promising candidates for the OER catalysts owing to their intrinsic activity and stability in alkaline conditions [2]. While individual Ni and Fe oxides possess poor conductivity and low OER activity, their integration yields a synergistic effect that significantly enhances both charge transport and catalytic efficiency [6, 7]. This synergy arises from the formation of Ni─O─Fe linkages, which promote electron redistribution and reduce the energy barrier for OER intermediates, even outperforming noble metal‐based catalysts in some cases [8]. In addition, Ni‐Fe oxides cost only a fraction of the IrO_x_ catalysts, approximately 50 times cheaper, [9] making them highly attractive for scalable water electrolysis applications [6, 10]. Nevertheless, despite these benefits, several challenges remain unresolved for the practical application of Ni‐Fe oxide catalysts.

Many of the Ni‐Fe oxide catalysts are conventionally synthesized as powders via ex situ fabrication methods, such as co‐precipitation, and these particles are subsequently coated onto diffusion layers. However, this approach frequently leads to critical delamination problems under anodic operating conditions. Because the individual catalyst particles are physically coated onto the substrates, they feature weak integration and structural discontinuity at the interface [5, 8, 11, 12, 13]. Even with binder polymers, the insufficient adhesion between the catalysts and the substrate makes the catalysts vulnerable to the mechanical stress from massive gas evolution [5, 14]. Since AEMWE can operate at high current densities reaching 5–6 A cm^−2^, these challenges can become even more severe.

To overcome these challenges, binder‐free, self‐supporting integrated electrodes, achieved by direct growth of nanostructured electrocatalysts on diffusion layers, have been proposed. Among the available techniques, hydrothermal synthesis and electrodeposition are considered two representative methods. The hydrothermal synthesis facilitates ion dissolution and subsequent reprecipitation of Ni and Fe species into targeted phases, including Ni‐Fe sulfides, selenides, and layered double hydroxides [15, 16]. Despite its simplicity and controllability, the hydrothermal process inherently suffers from limited scalability and long synthesis durations, as reactions occur within sealed autoclaves under high temperature and pressure. Alternatively, electrodeposition is carried out by applying controlled potentials or currents to induce the nucleation and growth of catalyst components directly on the electrode surface. This method enables precise control over film morphology, thickness, and composition—often surpassing the level of control achievable by hydrothermal synthesis [17]. Despite its advantages, electrodeposition also faces limitations in scalability due to difficulties in ensuring uniform film thickness and composition over large areas, as well as the increased cost and complexity of electrolyte management at scale [18]. Furthermore, even integrated electrodes with strong catalyst‐substrate bonding can degrade under high‐current density operations, as continuous stress from vigorous oxygen bubble evolution may cause partial delamination [19]. These challenges highlight the need for an integrated, operando‐formed catalyst architecture that exhibits strong interfacial anchoring and structural stability for enhanced durability.

In this study, we present an operando strategy to form an integrated Ni–Fe oxyhydroxide ((Fe, Ni)OOH) anode by converting bare Ni foam (NF) directly inside a practical AEMWE device. We leverage an electrochemical Fe doping strategy that has been often suggested in previous studies [20, 21, 22]. These studies, however, primarily focused on elucidating the mechanism of the dynamic Fe incorporation, detailed material characterization, and the origin of enhanced intrinsic OER kinetics. Here, we instead demonstrate how this strategy can be effectively implemented at the device level. In our approach, Ni–Fe oxyhydroxide catalysts are synthesized inside the device during the break‐in process by introducing only a trace amount of Fe species into the anode electrolyte. Because this operando fabrication does not require any separate electrode preparation steps, it is distinct from conventional in situ approaches, which still rely on hydrothermal synthesis or electrodeposition prior to assembly into the electrolyzer [23, 24]. The resulting integrated electrode possesses a defect‐rich (Fe, Ni)OOH structure characterized by a highly porous architecture with a thickness of several hundred nanometers. The (Fe, Ni)OOH‐NF electrode showcases excellent performance and durability in the AEMWE devices, achieving a current density of 2.8 A cm^−2^ at 1.8 V and remarkable stability over 7000 h in a two‐cell stack. These findings highlight that the operando electrochemical activation of bare Ni electrodes into the Ni‐Fe oxyhydroxide catalysts offers a simple route to realizing scalable, cost‐effective, high‐performance anodes for AEMWEs.

## 2 Results and Discussion

### 2.1 Operando Electrochemical Synthesis and Structural Characterization of (Fe, Ni)OOH‐NF

**FIGURE 1:**
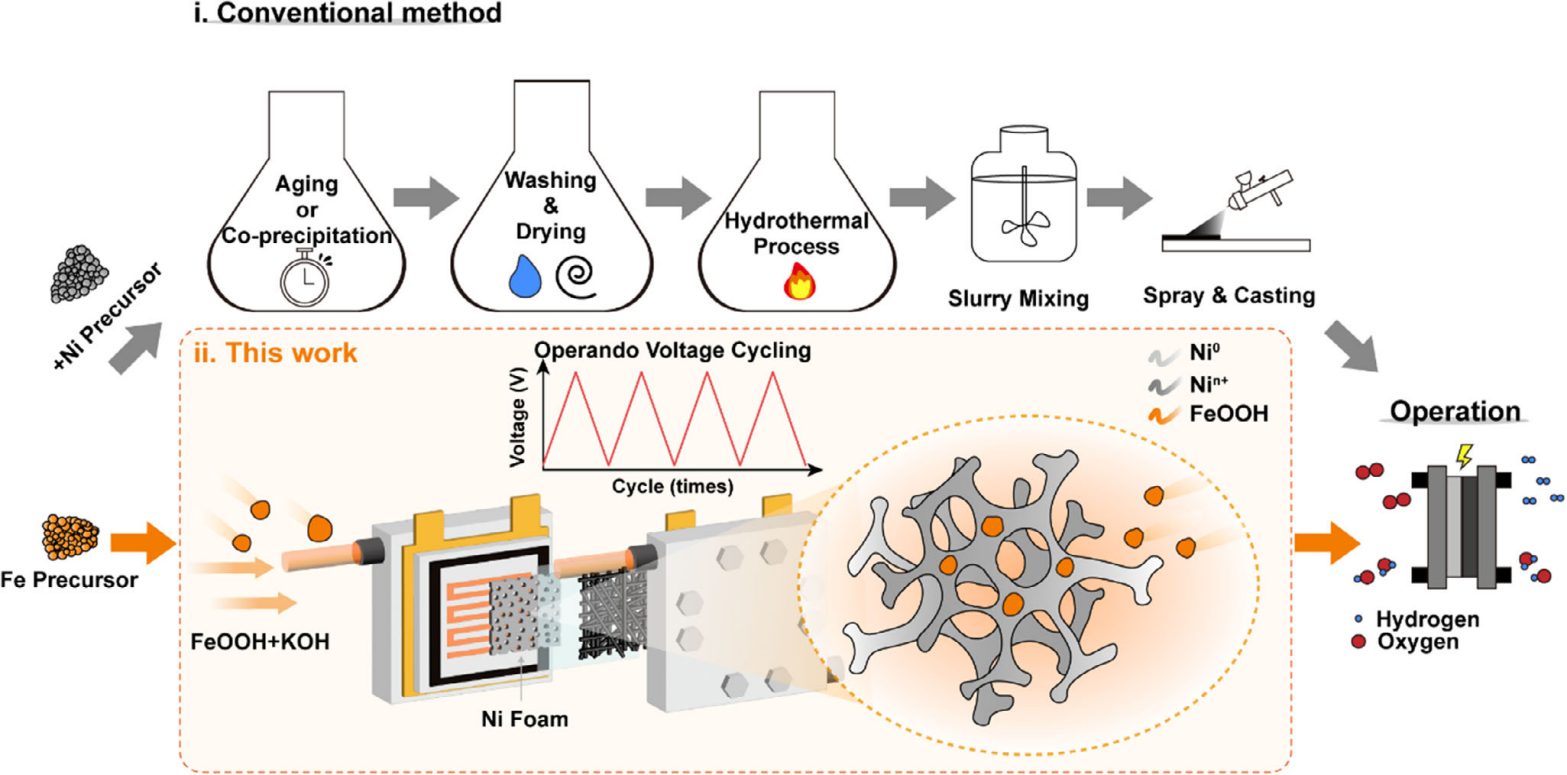
Schematic illustration of the fabrication processes for (i) conventional catalysts and (ii) operando‐synthesized (Fe, Ni)OOH‐NF electrode.

In conventional approaches, catalysts are typically synthesized as powders, mixed with binders, and then coated onto conductive substrates to prepare an AEMWE anode (upper illustrations in Figure 1). However, this method not only requires multiple manufacturing steps that significantly increase cost, but also results in insufficient interfacial contact, limited mechanical stability, and poor catalyst utilization within the electrode. To overcome these challenges, we present a chemically integrated (Fe, Ni)OOH electrode, in which the catalysts are developed directly on the Ni foam substrate during electrolysis. We leveraged a simple operando electrochemical activation method (bottom in Figure 1) and employed an Fe additive in the KOH electrolyte to enhance the electrocatalytic activity of the electrode. The major advantage of this method is that a pristine Ni foam can be instantly transformed into the (Fe, Ni)OOH electrode in a practical electrolysis device during the break‐in process, not requiring additional electrode manufacturing processes. Moreover, because this method enables chemical linkages between catalysts and substrate, the electrode is not vulnerable to delamination problems, providing high endurance in the water electrolysis anode.

As detailed in the experimental section, we selected FeCl_2_ as the activator and added 10 ppm into a 1 m KOH electrolyte, which is just a trace amount compared to the dominant KOH solutes but slightly higher than typical Fe concentrations in tap water (∼0.3 ppm). The added FeCl_2_ was transformed into brown flocculates in the electrolyte (Figure S1). The XRD analysis (Figure S2) revealed that they are predominantly composed of FeOOH phases, accompanied by minor amounts of Fe_2_O_3_ and Fe(OH)_3_, indicating that the initial Fe(II) species rapidly oxidized to Fe(III) in the KOH environment. Crystallite‐size evaluation using Scherrer and Williamson–Hall methods (Figure S3), together with DLS analysis (Figure S4), further confirmed that the dispersed flocculates are predominantly submicron in size and consist of nanoscale crystalline domains.

The Fe‐containing electrolyte was circulated through the anode compartment of an electrolyzer device, where bare Ni foam was employed, and voltage cycling was applied over a potential range of 1.54–1.76 V (Figure S5). This voltage range was selected to promote the formation of high‐valent Ni species and induce OER at the anode, thereby generating a highly roughened surface structure while avoiding excessively high potentials that could cause severe Ni corrosion. Notably, the Fe activators were largely confined to the anode compartment during the activation process and showed negligible interaction with other cell components, including the membrane and the cathode. Accordingly, no measurable changes in membrane or cathode performance were observed, as discussed in Note S2. The samples were named after the number of cycles; VC‐0, VC‐50, and VC‐800 NF denote the bare Ni foam and electrodes subjected to 50 and 800 cycles, respectively.

As discussed in the following sections, this simple and practical process allows direct formation of a porous and conductive Ni–O–Fe structure on the substrate, resulting in high activity for OER and enhanced electron conductivity compared to the pristine NF. In addition, the catalysts were tightly integrated on the Ni foam substrate, remarkably enhancing the longevity of the electrode even without using binders. Comparing with the previously reported binder‐free synthesis methods, such as hydrothermal synthesis or electrodeposition, which require ex situ catalyst preparation followed by electrolyzer assembly, the present approach achieves catalyst formation directly within the AEMWE devices during the electrochemical break‐in process using only a small amount of Fe in the electrolyte. This process‐integrated, one‐step strategy substantially reduced electrode fabrication steps, highlighting its potential for scalable manufacturing and reduced CAPEX.

**FIGURE 2:**
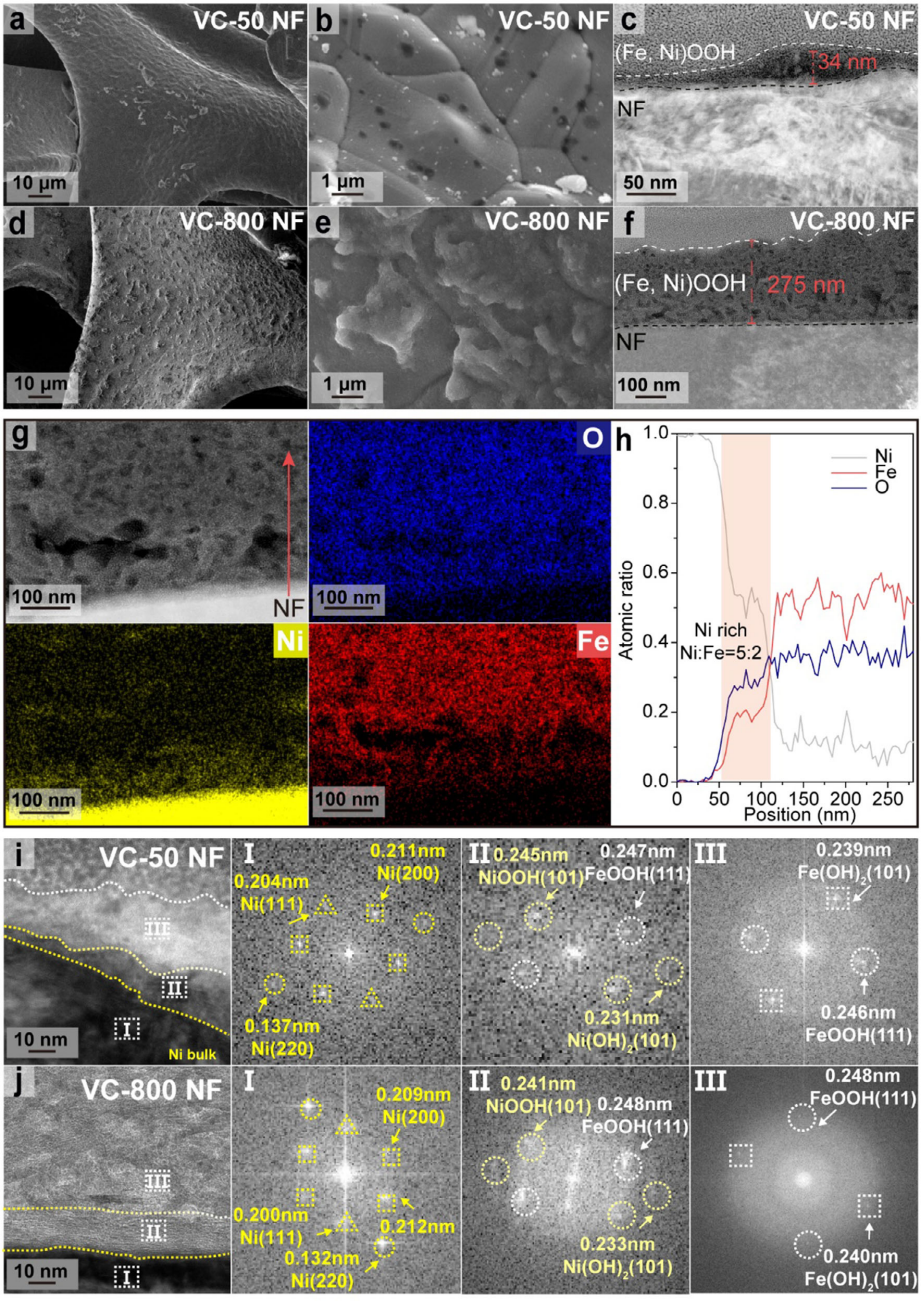
(a–f) Microscopical analysis of (a–c) VC‐50 NF and (d–f) VC‐800 NF electrodes. Surface SEM images at (a, d) low and (b, e) high magnifications. (c, f) Cross‐sectional TEM images. (g) STEM‐EDS elemental mapping for VC‐800 NF. (h) Atomic distribution across the VC‐800 NF obtained from EDS line‐scan analysis. (i, j) HR‐TEM images of (i) VC‐50 NF and (j) VC‐800 NF. Corresponding FFT patterns of the bulk Ni foam (I) and catalyst layers (II and III).

The electrochemically prepared electrode exhibited a brown color (Figure S9), consistant with the color of the Fe flocculates in the electrolyte (Figure S1). The color on the externally accessible surface of the 3D Ni foam remained intact even after vigorous cleaning with water or wiping with tissue. In addition, the brown layer was not detached even after sonication in water, implying tight integration of Fe species on the surface. This interpretation was further supported by post‐sonication SEM and XPS analyses, which showed that the electrode retained its highly rough surface texture without noticeable morphological degradation (Figure S10a,b) and exhibited virtually no change in surface composition after ultrasonication (Figure S11a,b). This is in stark contrast to the powder and binder‐based electrodes, the catalysts of which were easily delaminated from the substrate, as described in the SEM images (Figure S10c,d). Figure 2 provides an in‐depth investigation of the surface morphology and composition of the electrodes using SEM and TEM analyses. We investigated two electrode samples after 50 and 800 cycles to observe progressive growth of the catalyst on the surface.

SEM and TEM analysis in Figure 2a–f and Figure S9 revealed that the voltage cycling induced nanoscale roughening on the Ni foam surface, which is intensified at an increasing cycle number. While the pristine Ni foam featured a flat surface with only a few micrometers of cracks (Figure S12), the VC‐50 NF exhibited submicron‐scale particles and slight textures on its surface (Figure 2a,b). As shown in Figure 2d,e, both the particles and textures became more pronounced on the VC‐800 NF surface. The deposited particles could be easily distinguished from the cross‐section analysis (Figure 2c,f); the STEM images display dark nanostructures developed on the bright Ni foam substrate. The deposition was relatively thin and compact for VC‐50 NF (Figure 2c) because of the limited growth at the low cycle numbers, but it became significantly thicker (250–300 nm) on the VC‐800 NF electrode (Figure 2f), indicating pronounced structural development after the extended cycling. Notably, the textured surface on the VC‐800 NF exhibited nanoporous structures with increased surface exposure, which are expected to contribute to catalytic activity.

The elemental composition of the developed structure was investigated with EDS elemental analysis (Figure 2g; Figures S13 and S14). The surface mapping in Figure S14 confirmed the presence of Fe species on both VC‐50 and VC‐800 NF samples. While VC‐50 NF exhibited a discontinuous and particulate Fe distribution on the surface, that on the VC‐800 NF surface was generally uniform, suggesting that extended voltage cycling led to continuous Fe growth throughout the Ni foam. EDS analysis of the cross‐section STEM images (Figure 2g,h; Figure S13) further confirmed the depth‐dependent elemental distribution. For both VC‐50 and VC‐800 NF, oxygen was dominantly distributed on the Ni foam surface, indicating the formation of oxide phases. However, the Fe and Ni distributions differed markedly with the number of voltage cycles. In the case of VC‐50 NF (Figure S13), a broad layer with an approximate thickness of ∼30 nm was observed, exhibiting a Ni:Fe atomic ratio of ∼ 4:1, indicative of limited Fe incorporation. In contrast, VC‐800 NF displayed two distinct layers. Quantitative line mapping analysis revealed that the first 50 nm layer was enriched in Ni with an atomic ratio of 5:2 for Ni:Fe. But, in the outer layer, the Ni fraction became only one‐fifth of Fe. Despite the depth‐dependent elemental heterogeneity, the cross‐sectional analysis revealed seamlessly integrated Ni/Fe composite layers on the Ni substrate, thereby enhancing the mechanical robustness and durability of the electrode.

To further characterize the Ni and Fe oxides, high‐resolution TEM and FFT analyses were carried out on different regions of the deposition (Figure 2i,j). In both VC‐50 NF and VC‐800 NF, there existed three distinct layers: the Ni substrate, Ni‐rich inner layer, and Fe‐rich outer layer. The bulk Ni foam substrate (Region I) featured a well‐ordered crystalline structure, as expected; the FFT obviously produced the patterns of metallic Ni, including (111), (200), and (220) planes, which correspond to those of Ni (JCPDS no. 65–2865) [9]. The patterns became unclear as the oxide grew, and the outermost layers were almost amorphous with weak crystalline signals. The inner layers of both VC‐50 and VC‐800 NF (Region II) manifested mixed patterns of NiOOH, [25] Ni(OH)_2_, [26] and FeOOH phases [27]. In the outermost region (Region III), only the Fe patterns of FeOOH (111) and Fe(OH)_2_ (101) appeared, aligned with the line‐mapping profile, but the signals were mostly amorphous, signifying the disordered construction of composite oxides [28].

**FIGURE 3:**
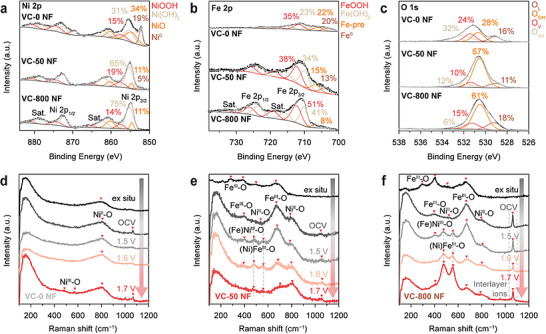
Material characterization of (Fe, Ni)OOH‐NF after the operando catalyst growth. (a–c) High‐resolution XPS analysis and quantification of (a) Ni 2p, (b) Fe 2p, and (c) O 1s spectra for VC‐0, VC‐50, and VC‐800 NF. (d–f) Ex situ and in situ Raman analysis at OCV (∼1 V), 1.5, 1.6 V to 1.7 V vs. RHE for (d) VC‐0 NF, (e) VC‐50 NF, and (f) VC‐800 NF.

High‐resolution XPS analysis in Figure 3a–c further provides the chemical state information of the deposited materials [10, 29, 30, 31]. According to the Ni 2p spectra (Figure 3a), the pristine Ni foam contained metallic Ni and oxidized species. The oxidized components were mostly Ni(II) states of NiO and Ni(OH)_2_, which are attributable to the native oxide layer. After 50 cycles of voltage cycling, the metallic Ni and NiO signals diminished, while the fractions of Ni(OH)_2_ and NiOOH components became dominant to 65% and 19%, respectively, indicating activation toward an (oxy)hydroxide‐rich surface. In parallel, the trace amount of Fe content (2.1 at. %) in the bare Ni foam increased to 6.4 at. % after the voltage‐cycling (Table S1 and Figure S15). The Fe 2p spectra (Figure 3b) indicated that the increased Fe components are mostly the FeOOH (38%) and Fe(OH)_2_ (34%) species, which is in good agreement with the FFT result. The O 1s spectra (Figure 3c) corroborated these transitions, showing a remarkable increase in O_OH_ contributions (67%) compared to that in the pristine surface (28%). The prolonged voltage‐cycling (VC‐800 NF) intensified this transition; metallic Ni was fully suppressed, and the surface was stabilized into a Ni(OH)_2_‐dominant phase. Concomitantly, the atomic fraction of Fe further increased to 11.3 at.%, while the FeOOH signal became even stronger (51%), confirming the formation of a Fe^3+^‐rich structure. Overall, the FFT and XPS analyses converge on the conclusion that the developed oxide layers are mostly in Ni(OH)_2_ and FeOOH structure, while the composites are highly amorphous with multiple defects.

Nevertheless, it should be noted that the material's properties under actual operating conditions often differ from those observed during ex situ characterization. To narrow this gap, we performed in situ Raman analysis under OER‐active potentials (1.5–1.7 V vs. RHE) in the KOH electrolyte environment (Figure 3d–f). The Raman data of the three electrode samples at open‐circuit voltage (OCV) coincided with the ex situ analyses. The pristine VC‐0 NF (Figure 3d) displayed a weak and broad Ni(II)–O vibration peak at 800 cm^−1^ due to the native NiO film, [32] and the (Fe, Ni)OOH‐NF electrodes (Figure 3e,f) produced Fe(III)–O and Ni(II)–O signals; the Fe peaks appeared at 390 and 700 cm^−1^, [33, 34, 35] and the Ni signals were detected at 530 and 800 cm^−1^ [36]. These are in line with the XPS and FFT analyses that also demonstrated the presence of FeOOH and Ni(OH)_2_ components for both the VC‐50 and VC‐800 NF electrodes.

When applying the anodic potentials, however, the signals changed significantly, and their variation was dependent on the samples. In the case of VC‐0 NF, applying potential did not make meaningful differences in the spectra, only displaying the invariant Ni(II)–O peak at 800 cm^−1^. By contrast, applying potential did change the spectra for the electrochemically treated electrodes. For the VC‐50 NF, an increasing potential progressively weakened the original bands, especially the Fe(III)‐O peak at 700 cm^−1^, and new signals appeared at 480 and 560 cm^−1^, which were ascribed to (Fe, Ni)OOH components; [37] more specifically, the former peak corresponds to the (Fe)Ni(III)–O signal, while the latter is that of (Ni)Fe(III)–O. These phenomena were more obvious in the VC‐800 NF electrode. Upon applying potentials of 1.5–1.7 V vs. RHE, the (Fe, Ni)OOH peaks at 480 and 560 cm^−1^ were dramatically intensified, while the Fe(III)–O peak gradually disappeared. Because these new features did not appear on the pristine Ni substrate but became apparent at the high‐Fe‐loading electrode, we inferred that the abundant Fe components, along with the defective oxygen bonding, enabled the stable and highly oxidized Ni species at the high potentials. The present electrochemical Fe incorporation thus not only accelerates the formation of mixed Ni–Fe oxyhydroxides but also promotes electronic coupling between Ni and Fe, enabling charge redistribution that stabilizes the active Ni^3+^ state during OER operation [38, 39]. While the above discussion remains qualitative, the observed spectroscopic trends are consistent with prior theoretical insights into Ni–Fe oxyhydroxides, suggesting a similar underlying OER mechanism. A detailed electronic‐structure analysis of the Ni–O–Fe framework via DFT and XAFS will be pursued in future work but is beyond the scope of this device‐focused study.

Collectively, the Raman spectra provide important complementary insights to the ex situ characterizations, revealing the activation pathway of the Ni foam: VC‐0 NF remains catalytically inert under applied potentials, VC‐50 NF undergoes progressive restructuring into active Ni‐Fe oxyhydroxides but with a compact structure, and VC‐800 NF with plentiful Ni─O─Fe bonding effectively stabilizes abundant Ni^3+^/Fe^3+^ oxyhydroxide species along with a high porosity. These analyses validate the efficacy of our operando activation methods, in which the electrochemical Fe‐doping could achieve a (Fe, Ni)OOH framework that is active for the alkaline oxygen evolution reaction.

**FIGURE 4:**
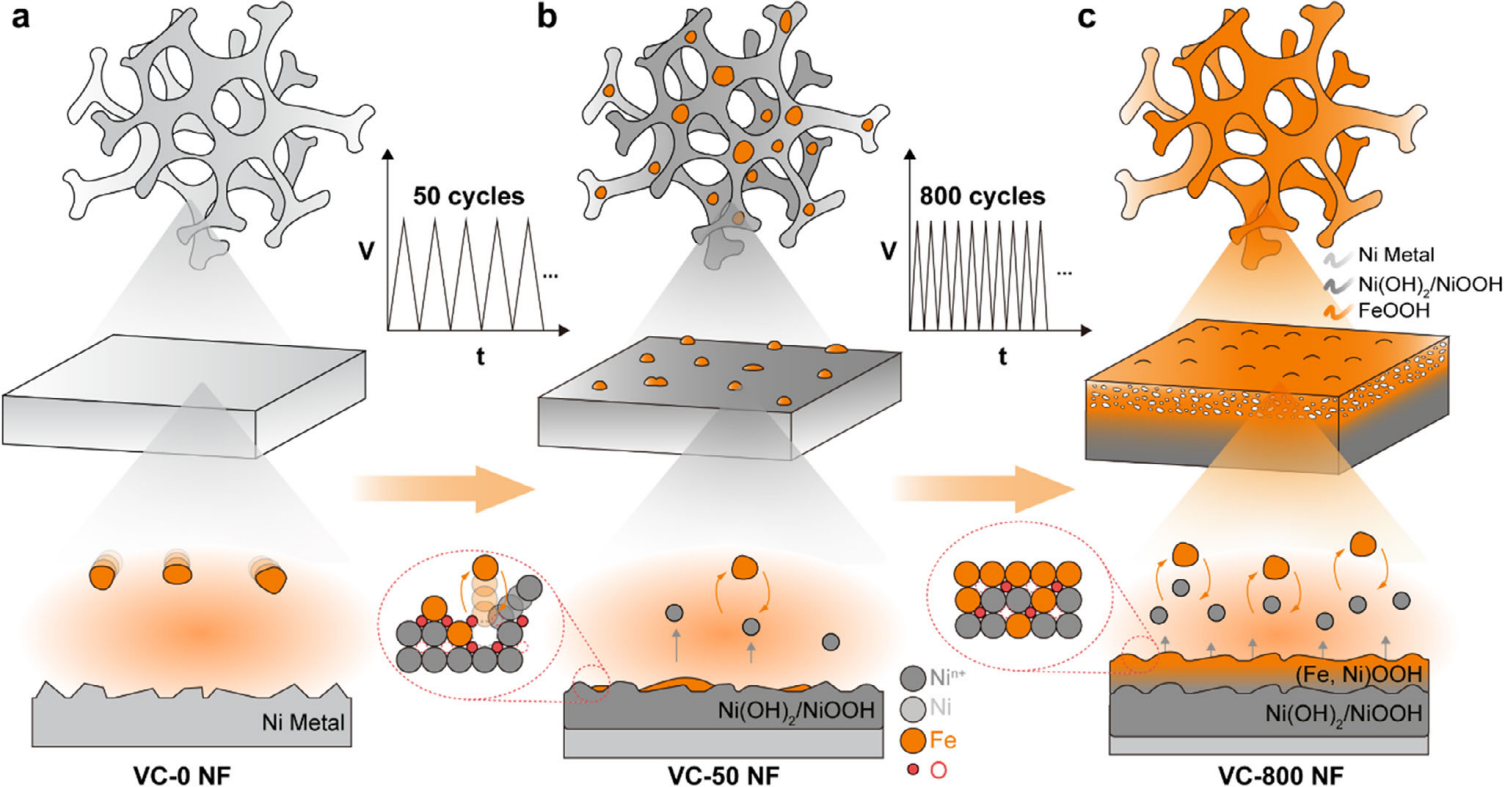
(a–c) Schematic depiction of the morphological changes in (Fe, Ni)OOH‐NF and the dynamic compositional evolution of Ni and Fe during voltage‐cycling. (a) VC‐0 NF, (b) VC‐50 NF, and (c) VC‐800 NF.

Based on the morphological, compositional, and structural analyses described above, the mechanistic pathways of voltage‐induced catalyst fabrication are summarized in Figure 4. The initial Ni foam surface, composed primarily of Ni metal and NiO, is electrochemically inactive for OER. Upon exposure to a Fe‐containing electrolyte, Fe ions and flocculates physically adsorb onto the Ni surface. During the voltage cycling, partial oxidation of the Ni substrate occurs, forming a Ni (oxy)hydroxide matrix with a small amount of Fe particles near the surface (VC‐50 NF). As the cycling goes on, FeOOH gradually incorporates into the Ni (oxy)hydroxide matrix, leading to a lattice reorganization and forming an intermixed (Fe, Ni)OOH layer.

Inductively coupled plasma mass spectrometer (ICP‐MS) analysis (Figure S16) confirmed that Ni content in the pristine electrolyte was negligible, and we inferred that the Ni of the (Fe, Ni)OOH structure is solely from the Ni foam substrate. We speculate that Ni ions dissolved from the substrate during anodic scans, and most of them were redeposited on the substrate in the opposite direction, together with the Fe components in the electrolyte. The reduced concentration of Fe species in the electrolyte after the cycling supports this speculation; nearly 20% of the Fe inputs disappeared after the voltage cycling. This progressive dissolution and redeposition yielded a nanoporous catalyst structure with a Ni:Fe atomic ratio of approximately 5:2 near the substrate. Above the 50 nm thick layer, however, as the Ni ion supply was reduced due to limited diffusion from the substrate, deposition of the floating Fe components became predominant and resulted in the Fe‐rich outer layer. Consequently, the voltage‐cycling‐induced Fe doping provides a dual benefit of porous structural reconstruction and a high population of catalytically active Ni sites.

### 2.2 (Fe, Ni)OOH‐NF Electrocatalytic Performance

**FIGURE 5:**
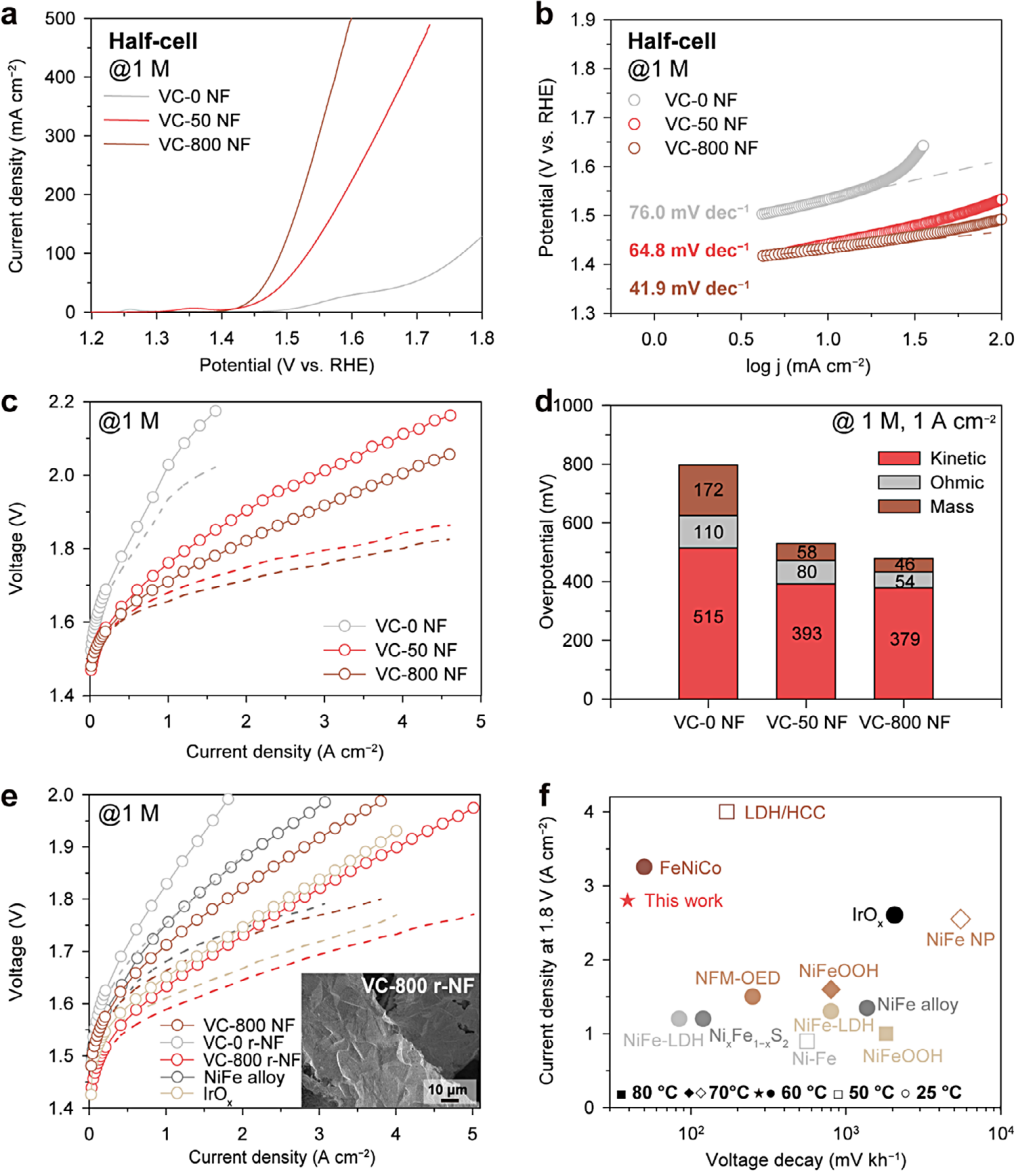
Electrochemical analysis of VC‐0 NF, VC‐50 NF, and VC‐800 NF in 1 M KOH in half‐cell and single‐cell. (a) OER polarization curves of the electrodes in a half‐cell configuration at room temperature. (b) Tafel plots derived from the polarization curves in (a). (c) IV polarization curves of the electrodes in a single‐cell AEMWE in 1 m KOH at 60°C. Dashed curves indicate iR‐corrected polarization curves. (d) Overpotential breakdown at 1 A cm^−2^ from the polarization curves shown in (c). (e) IV polarization curves of the (Fe, Ni)OOH electrodes in 1 m KOH at 60°C compared with the IrO_x_ and NiFe alloy‐coated electrode. VC‐800 r‐NF is the electrochemically Fe‐doped electrode using an acid‐treated Ni foam. (f) Single‐cell performances of the state‐of‐the‐art catalysts in 1 m KOH, depending on the voltage decay, and their comparison with (Fe, Ni)OOH‐NF [13, 40, 41, 42, 43, 44, 45, 46, 47].

To verify whether these structural and chemical evolutions resulted in an enhanced intrinsic OER performance, the VC‐0, VC‐50, and VC‐800 NF electrodes were electrochemically evaluated at both half‐cell and single‐cell levels. The (Fe, Ni)OOH electrodes that have been prepared in single‐cell devices were also analyzed in a half‐cell system, in order to probe the kinetic effect separately. As shown in Figure 5a, OER kinetics improved progressively with increasing voltage cycling, VC‐800 NF exhibiting the highest activity. The overpotential at 20 mA cm^−2^ decreased from 334 mV (VC‐0 NF) to 232 mV (VC‐50 NF), and further to 215 mV (VC‐800 NF). This enhancement underscores the beneficial role of the (Fe, Ni)OOH catalysts on the OER kinetics. The Nyquist plot in Figure S17a corroborates the improved charge‐transfer kinetics. Correspondingly, the Tafel plot (Figure 5b) revealed significant reductions of kinetic overpotential for the Fe‐doped electrodes, along with the slope decrease from 76.0 mV dec^−1^ for VC‐0 NF to 41.9 mV dec^−1^ for VC‐800 NF. These indicate that the electrochemical reconstruction has accelerated the OER kinetics by means of a mechanism change and expanded active surface area. Cyclic voltammetry (CV) plot also showed a gradual increase in the Ni^2+^/Ni^3+^ redox peak with the cycle number (Figure S17b), representing the larger number of electrochemically accessible sites. This trend is consistent with the electrochemical surface accessibility inferred from double‐layer capacitances (Figure S18a–c,f); a pronounced increase was observed in capacitive response from VC‐0 to VC‐800 NF, following the same trend as the polarization performance. We attribute the enhanced OER performance to the increased surface area and the high density of OER‐active species. In other words, the improvement arises from the combined effects of porous architecture, observed in the SEM and TEM images, and enlarged population of Ni(III) species, revealed by the in situ Raman spectra.

The kinetic effects were also apparent in the single‐cell polarization (Figure 5c). The Fe‐doped electrodes produced a remarkably high current density of 2 A cm^−2^ at 1.82 V, which is 5 times that of the pristine Ni foam (0.5 A cm^−2^). As expected, a substantial decrease in the charge‐transfer resistance (R_ct_) was the primary reason for the enhancement. This was verified from the iR‐corrected polarization curves (Figure 5c) and subsequent overpotential breakdown (Figure 5d). In addition, the R_ct_ of the pristine Ni foam (0.18 Ohm cm^2^) was reduced to 0.06 Ohm cm^2^ after the Fe doping, according to the impedance analysis (Figure S19). It should be noted that the increased catalyst surface facilitated the material transport of oxygen and water into each active site, thereby lowering the transport overpotential, as shown in Figure 5d. Interestingly, the Fe doping also accompanied a reduction of ohmic overpotential. Destruction of the poorly conductive Ni native oxide and transformation into the conductive (Fe, Ni)OOH structure accounts for this reduction [48]. This indicates that the chemically integrated catalyst‐PTL structure mitigated the contact resistance, thereby enhancing the single‐cell performance by 54 mV at 1 A cm^−2^ (Figure 5d).

The integrated (Fe, Ni)OOH‐NF electrode was benchmarked against IrO_x_ and the NiFe alloy anode. High loading of commercial IrO_x_ and NiFe alloy catalysts, mixed with an ionomer, were spray‐coated onto a substrate (see Section 4), which represent typical OER anode benchmarks [5, 49, 50]. SEM images (Figure S20) show a porous and uniformly coated catalyst layer composed of few‐nanometer‐sized particles. Although the (Fe, Ni)OOH catalyst substantially enhanced the single‐cell performance, the current density of VC‐800 NF slightly lagged behind that of the IrO_x_ electrode (Figure 5e). We ascribe this to the smaller surface area of the (Fe, Ni) OOH layer compared with the nanometer‐scale IrO_x_ particles, as evidenced by the BET analysis in Figure S21a. This suggests that further increases in the electrochemically active surface area could improve performance. It is worth noting that the catalytic site density of the (Fe, Ni)OOH‐NF electrode can be readily expanded by engineering the surface morphology of the Ni substrate, for example, by employing Ni substrates with a smaller particle size or by surface roughening.

In this study, the expanded surface area was achieved through a simple oxalic acid treatment of the pristine Ni foam substrate. The roughened Ni foam (r‐NF) substantially provided the available surface area for catalyst growth (inset SEM image of Figure 5e), as evidenced by BET analysis (Figure S21a); the surface area of VC‐0 r‐NF (0.489 m^2^ g^−1^) was 54 times larger than that of the original VC‐0 NF (0.009 m^2^ g^−1^). Consistently, ECSA‐related measurements further showed that the pretreated substrate developed a remarkably larger ECSA (Figure S18d–f) without noticeable changes in the overall surface chemical composition, as supported by Figure S22a,b. As a result, surface roughening led to a marked performance improvement without altering the operando catalyst fabrication process, even outperforming the IrO_x_ electrode (Figure 5e). Overpotential breakdown (Figure S21b) of the VC‐800 r‐NF polarization further demonstrated a marked decrease in kinetic overpotential, verifying its improved charge‐transfer kinetics. Notably, VC‐800 r‐NF achieved a current density of 2.8 A cm^−2^ at 1.8 V, surpassing the state‐of‐the‐art, non‐PGM OER catalysts while requiring a significantly lower catalyst loading (Figure 5f). The OER Faradaic efficiency under the same single‐cell operating conditions was independently measured to be ∼99.9% via O_2_ collection by water displacement, confirming that the applied current was predominantly utilized for oxygen evolution (Figure S23a,b). Collectively, these results validate that our approach enables the operando formation of a highly active, conductive, and compositionally intermixed (Fe, Ni)OOH catalyst layer, resulting in superior catalytic activity, reduced charge‐transfer resistance, and excellent practical performance.

### 2.3 (Fe, Ni)OOH‐NF Electrocatalytic Stability

**FIGURE 6:**
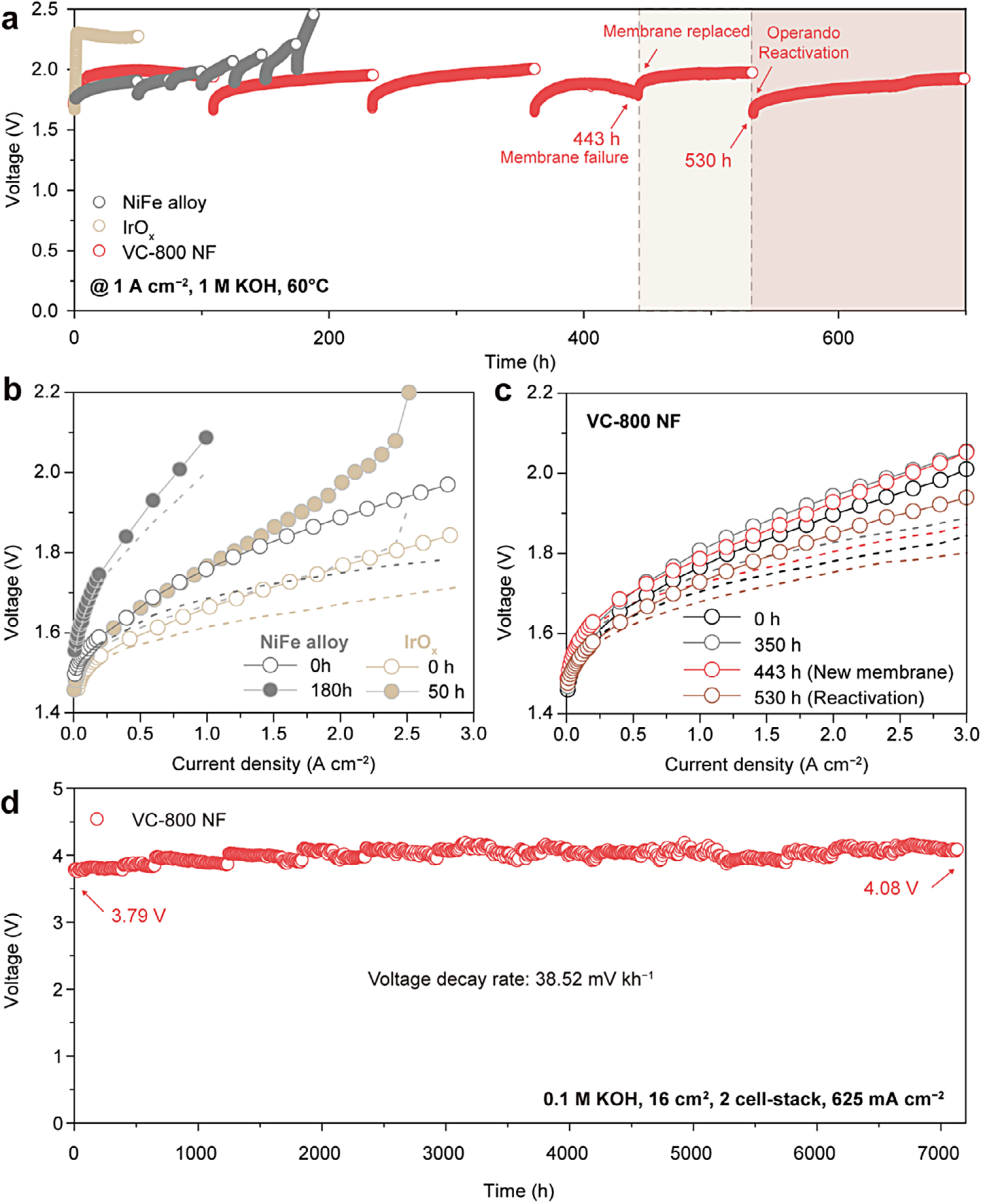
Long‐term stability test of the (Fe, Ni)OOH‐NF single cells. (a) Chrono‐potentiometry (CP) data of VC‐800 NF, IrO_x_, and NiFe alloy particles measured at a constant current density of 1 A cm^−2^ in 1 m KOH at 60°C. (b, c) IV Polarization curves of the (b) IrO_x_, NiFe alloy, and (c) VC‐800 NF cells before and after the CP test. For VC‐800 NF, the membrane replacement and reactivation processes are indicated by different color shades. Dashed curves indicate iR‐corrected polarization curves. (d) Voltage profile of the 2‐cell stack AEMWE using the VC‐800 NF anodes, operated for 7130 h in 0.1 m KOH at 625 mA cm^−2^ and unheated operation.

Figure 6 further examines the long‐term durability of the (Fe, Ni)OOH‐NF electrodes at both single‐cell and stack levels. The single‐cell test was performed at 1 A cm^−2^ under the practically relevant conditions (1 M KOH, 60°C) for the VC‐800 NF electrode, monitoring the voltage profile (Figure 6a) along with polarization (Figure 6b) and impedance diagnostics. VC‐800 r‐NF, which was included to assess the maximum initial performance achievable by substrate pretreatment, also exhibited stable operation throughout without a severe voltage increase (Figure S24). By contrast, the IrO_x_/PTL cell exhibited early‐stage degradation, characterized by an abrupt voltage increase within 50 h, which was attributed to interfacial delamination [5]. These differences are substantiated by the polarization curves measured before and after the CP test, shown in Figure 6b,c. The IrO_x_ cell lost its catalytic activity after 50 h (Figure 6b), due to delamination and loss of the catalysts from the substrate. This rapid degradation is also consistent with previous reports on conventional coated IrO_x_ electrodes, in which sustained oxygen evolution induces catalyst‐layer detachment and interfacial instability under operating conditions [13, 51, 52, 53]. The NiFe alloy particle electrode also showed inferior durability, but with a different temporal profile. Specifically, it exhibited a gradual voltage increase over the first 100 h, followed by a much sharper rise near 180 h. This behavior is also consistent with the ultrasonication results, where the NiFe alloy electrode showed partial detachment after treatment, in contrast to the negligible surface change observed for VC‐800 NF. These observations suggest that the degradation of the NiFe alloy particle electrode is also associated with weak interfacial adhesion and catalyst‐layer delamination, which is further reflected in the polarization curves measured before and after the CP test (Figure 6b,c). By comparison, the VC‐800 NF cell retained a stable polarization even after 350 h of operation (Figure 6c). As discussed earlier, this pronounced difference in durability arises from the distinct electrode architectures, namely, binder‐containing particulate catalyst layer versus the binder‐free, integrated electrode configuration.

The VC‐800 NF anode maintained stable operation over extended periods; however, gradual voltage drops emerged at approximately 400–443 h, accompanied by short‐circuit events in the OER‐inactive voltage region, indicating membrane failure. After replacing the failed membrane while retaining the same electrodes, the voltage profile stabilized, comparable to the polarization behavior before the membrane failure. Nonetheless, an increase in overpotential was observed from the polarization curve at 443 h, indicating that electrodes also degraded along with the membrane failure. To clarify the origin of electrode degradation, we quantified Fe loss by ICP–MS analysis for both the VC‐800 NF anode (Figure S25) and the anolyte (Figure S26) before and after 300 h of operation. The anode Fe loading decreased from 26.4 to 21.3 µg cm^−2^, corresponding to a net loss of 20.4 µg and a dissolution rate of 0.017 µg cm^−2^ h^−1^. The anolyte analysis aligns well with the electrode analysis; the Fe content increased by 35.5 µg in the anolyte. Together, these results confirm measurable Fe leaching during operation and its predominant accumulation in the electrolyte, and the values are within the range of those reported for Ni–Fe hydroxyoxide catalysts [22, 44]. This Fe loss is consistent with the observed increase in kinetic overpotential (Figure S27), supporting Fe depletion as a contributor to the degradation behavior. The agreement between electrode Fe loss and electrolyte Fe accumulation should be regarded as semi‐quantitative, as small discrepancies can arise from incomplete collection of redistributed Fe species, and possible retention at other system components.

We would like to emphasize that this degradation, however, can be effectively recovered by applying our activation protocol. At 530 h, we repeated the operando voltage‐cycling protocol identical to the initial fabrication procedure to intentionally reactivate the anode, which restored—and even enhanced—the performance beyond the initial level. This reactivation behavior suggests that the integrated (Fe, Ni)OOH anode underwent additional catalyst growth, whereby electrochemical cycling could recover intrinsic activity after prolonged operation; consistent with this interpretation, impedance analysis shows concurrent decreases in both ohmic resistance and charge‐transfer resistance (Figure S28a,b). This recoverable behavior is also supported by post‐mortem characterization of VC‐800 NF after single‐cell durability operation. As shown in Figure S29a,b, the rough, integrated surface morphology and the continuous Ni–Fe‐containing layer were retained without evident large‐scale delamination or structural collapse. Although XPS and ex situ Raman analyses (Figure S30a–d) revealed surface chemical reconfiguration accompanied by some Fe redistribution during operation, the post‐durability electrode still preserved the integrated mixed Ni–Fe oxyhydroxide‐derived surface character (Figure S31a,b). These observations suggest that durability operation leads primarily to compositional and chemical evolution of the active surface, rather than irreversible destruction of the integrated electrode architecture. Overall, the (Fe, Ni)OOH anode exhibits high durability over 500 h with a degradation rate of 45.10 mV kh^−1^, and importantly, its performance can be effectively regenerated via operando electrochemical reactivation using only a trace amount of Fe and simple voltage cycling. From a practical perspective, this regeneration does not necessarily require continuous Fe addition. These results further suggest that the proposed operando and integrated regeneration strategy can be translated into a practical maintenance protocol for durable AEMWE operation. From a system‐engineering perspective, the Fe dosage should be minimized to reduce unnecessary Fe accumulation, contamination risks, and other unintended side effects within the recirculating electrolyte loop. In the present work, effective reactivation was achieved with Fe concentrations at the tens‐of‐ppm level, indicating that only a small amount of Fe is required for catalyst maintenance. In practical operation, regeneration may be triggered either periodically or by a predefined performance criterion, such as a voltage increase at fixed current density or a detectable change in impedance response. Considering the degradation rate observed here (∼30–50 mV kh^−1^), a regeneration interval on the order of 1000–3000 h may serve as a reasonable engineering starting point before substantial performance loss accumulates. Nevertheless, the exact dosage, trigger threshold, and regeneration frequency will depend on the electrolyte inventory, recirculation design, current density, and target durability window of the specific system.

The device‐level behavior of VC‐800 NF was further demonstrated in a two‐cell AEMWE stack that sustained a stable voltage profile at 625 mA cm^−2^ for 7130 h (Figure 6d), exhibiting a voltage decay rate of 38.52 mV kh^−1^. Figure S32a compares the linear sweep voltammetry curves obtained before and after the durability test, showing only a modest upward shift at a given current density after long‐term operation. The impedance and overpotential analyses (Figure S32b,c) indicate that the observed performance degradation is mainly attributable to increased mass‐transport and activation polarizations, whereas the ohmic resistance remains essentially unchanged. This result suggests that ionic transport through the membrane was largely preserved during long‐term operation. Post‐mortem analyses indicate minimal metal crossover and cathode degradation: ICP–MS shows no meaningful change in Fe concentration of the cathode effluent (Figure S33a), and XPS/SEM‐EDS revealed no discernible Fe on the PtRu/C cathode with negligible morphological change (Figures S33b and S34a–d).

SEM analysis confirms that the anode maintains its rough morphology after the long‐term operation (Figure S35a,b), whereas TEM‐EDS results show compositional evolution, where the Fe‐rich outer gradient becomes uniform after 7000 h (Figure S36a,b), likely contributing to the observed polarization increase. Post‐durability XPS, ex situ Raman, and XRD analyses further show that the integrated VC‐800 NF surface underwent substantial surface reorganization during operation. Specifically, the Ni 2p, Fe 2p, and O 1s spectra indicate redistribution of Ni/Fe (oxy)hydroxide together with modification of the oxygen coordination environment (Figure S37). Consistently, XRD (Figure S38) shows weakening of the Fe‐ and NiO‐related diffraction features and a more pronounced NiOOH‐related signal after long‐term operation, in line with partial Fe dissolution/redistribution and progressive restructuring of the Ni‐containing surface. Importantly, these results indicate that, while the chemical states were slightly changed, the anode surface retained a mixed Ni–Fe oxyhydroxide‐derived character even after the long‐term operation. It should be noted that we intentionally tested the two‐cell stack under externally unheated conditions with 0.1 m KOH to minimize membrane‐driven failure modes; although the stack was expected to self‐heat (∼40°C) during operation, these conditions may have partially delayed certain degradation processes compared to harsher, industry‐relevant operation. Despite mild conditions, this result underscores the long‐term mechanical and electrochemical resilience of the operando‐formed (Fe, Ni)OOH layer, positioning it as a competitive alternative to noble metal catalysts for large‐scale AEMWE deployment.

Taken together, the above durability results provide the strengths and applicability of our operando electrode fabrication strategy in practical devices. Benchmarking against recent NiFe‐based AEMWE anodes (Table S2), our integrated anode exhibits a favorable performance–durability balance, combining a low catalyst loading (0.25 mg cm^−2^) with high current density at 1.8 V (2.8 A cm^−2^ at 60°C) and sustained stability (39 mV kh^−1^ over 7000 h). A materials‐cost analysis further indicates that the catalyst cost can be markedly reduced by the trace‐Fe operando approach (Table S3) [9, 54, 55, 56]. Finally, the manufacturability and scalability of the protocol are supported by the successful preparation of VC‐800 NF electrodes from 2 × 2 cm^2^ up to 30 × 20 cm^2^ using the same procedure (Figure S39).

## 3 Conclusions

This study demonstrated that operando electrochemical Fe doping enabled the direct formation of highly active, conductive, and durable (Fe, Ni)OOH catalyst layers on Ni foam. The resulting (Fe, Ni)OOH‐NF electrode exhibited exceptional electrocatalytic activity and stability, even outperforming the IrO_x_ electrode. The single‐cell achieved a current density of 2.8 A cm^−2^ at 1.8 V, and the two‐cell stack demonstrated remarkable stability over 7000 h, showing only a polarization loss of 10%. These results demonstrate the efficacy of our integrated electrode for interfacial robustness, high activity, and even electron conductivity. In particular, the exceptional performance and durability under practical operating conditions highlighted the feasibility of this approach for large‐scale AEMWE applications, providing an economically viable alternative to conventional electrode fabrication methods.

## 4 Experimental Section

### 4.1 Synthesis of QPC‐TMA

The ionomer material based on quaternized polycarbazole with anion‐conducting properties (poly(9‐(6‐(trimethylammoniumbromide)‐hexyl)‐9H‐carbazole‐co‐1,1,1‐trifluoroisopropane); QPC‐TMA) was prepared following a previously documented procedure involving poly(9‐(6‐bromohexyl)‐9H‐carbazole‐co‐1,1,1‐trifluoroisopropane) (PC‐Br) and a 45 wt.% trimethylamine solution [57].

### 4.2 Preparation and Characterization of the CLs

QPC‐TMA was uniformly dispersed at 10 wt.% in a 7:3 binary solvent of dimethyl sulfoxide (DMSO) and water using roller ball‐milling for 12 h. DMSO serves as an optimal solvent for dispersing QPC‐TMA into small sizes. IrO_x_ catalyst (Iridium (IV) oxide, Premion, 99.99%, Ir 84.5% min) and PtRu/C catalyst (29.8 wt.% Pt, 23.1 wt.% Ru, TEC61E54, Tanaka Kikinzoku Kogyo) were each homogeneously mixed with the QPC‐TMA dispersion by roller ball‐milling for 24 h. The solid content of the slurries was maintained at 10 wt.%. These catalyst slurries were sprayed using an airbrush (HP‐C Plus, Iwata) onto a platinum‐coated titanium porous transport layer (Pt‐Ti‐PTL, 2GDL6N‐025, Bekaert) for IrO_x_ and onto a carbon gas diffusion layer (GDL, JNT30A3, JNTG) for PtRu/C. The loadings of IrO_x_ and PtRu for the anode and cathode catalyst layers (CLs) were controlled to be 2.0 ± 0.01 and 0.4 ± 0.01 mg cm^−2^, respectively.

### 4.3 Operando Fe Integration on Ni Foam by Voltage Cycling

Ni foam (99.98%, TMAXCN) with dimensions of 320 ± 3 µm and 98% porosity was used. 10 ppm Fe from FeCl_2_ (Iron (II) chloride tetrahydrate, Sigma–Aldrich, 99%) was dissolved in deionized (DI) water and mixed with 1 m KOH (Potassium hydroxide, Sigma–Aldrich, 90%) at a mixing ratio of 0.0018 to 1 in weight. After the mixing, an orange‐colored Fe hydroxide suspension was formed. The suspension was circulated through the anode, individually at 60°C. Voltage cycling was conducted in a range of 1.54–1.76 V at a scan rate of 50 mV s^−1^. After 800 cycles of voltage‐cycling, the Ni foam exhibits a final mass increase of approximately 0.25 mg cm^−2^.

### 4.4 Electrochemical Analyses in Half‐Cell

A half‐cell test was performed using a three‐electrode cell (Pine Research, standard electrochemical cell, AKECLL2) and a VSP potentiostat system (Biologic). Electrochemical analyses were conducted in a 1 m KOH solution (Sigma–Aldrich, 99.99%) with N_2_ (99.99%) purging for 15 min at room temperature. A coiled Pt wire and Hg/HgO were used as the counter and reference electrodes, respectively. The Ni foam served as the working electrode for OER measurements, with an areal area of 1 cm^2^. Linear sweep voltammetry (LSV) was measured at a scan rate of 20 mV s^−1^ in nitrogen‐purged 99.99% 1 m KOH. Voltage cycling was executed at a scan rate of 20 mV s^−1^ within a voltage range of 1.54–1.76 V vs. RHE. Electrochemical impedance spectroscopy (EIS) for OER was conducted at 1.5 V vs. RHE over a frequency range of 100 kHz to 100 mHz with a 5 mV amplitude. Tafel plots were derived and fitted to the Tafel equation (η = b log j + a) in the linear portions at low overpotentials from the LSV data [58].

### 4.5 Preparation of Membrane Electrode Assemblies (MEAs), Single‐Cell and Two‐Cell Stack

The 30 ± 3 µm‐thick QPC‐TMA AEM (IEC = 2.4 mequiv g^−1^) was stored in 1 m KOH for 1 h to replace Br^−^ ions with OH^−^ ions before MEA fabrication. For single‐cell fabrication, the QPC‐TMA AEM was sandwiched between a CL‐coated substrate and GDL. The MEA was assembled with 230 µm‐thick gaskets and titanium and graphite flow fields at the anode and cathode, respectively. The active area of the electrode was 4 cm^2^, and the cell was compressed by eight bolts at 20 kgf cm. The Ni foam was immersed in 4 wt.% oxalic acid for 12 h to increase its surface area, producing oxalic acid‐treated rough Ni foam. The IrO_x_ catalyst for the control group was prepared using a 10 wt.% ionomer applied via the spray method onto a Pt‐coated PTL, and the NiFe alloy catalyst was prepared onto a nickel foam. The two‐cell stack had a 4 × 4 cm^2^ active area with a conventional 80 µm Piperion AEM, compressed with eight bolts at 30 kgf cm torque. All other conditions matched those of the single‐cell setup.

### 4.6 Single‐Cell Performance Tests and Electrochemical Analyses

All electrochemical analyses were performed using a high current potentiostat (HCP‐803, BioLogic). The *i*–*V* polarization curves were measured at 60°C with alkaline electrolyte circulation for the anode. Current densities were increased stepwise by 0.02 A cm^−2^ from 0 to 0.2 A cm^−2^, then by 0.2 A cm^−2^ until the cell voltage reached 2 V, maintaining each step for 2 min to indicate steady‐state current density values. EIS was conducted at 0.2 A cm^−2^ with a 10 mV amplitude in KOH aqueous solution, over a frequency range of 100 kHz to 100 mHz.

A long‐term durability test was performed by chronopotentiometry at a fixed current density of 1 A cm^−2^ with a 1 m KOH feed in a single cell. Roughened Ni foam (r‐NF) was prepared with aging in 10 wt.% oxalic acid for 12 h. In the stack, chronopotentiometry was executed with 0.1 m KOH at 625 mA cm^−2^, over a 4 × 4 cm^2^ area, at unheated operation.

### 4.7 Physical Characterizations

Surface images, energy‐dispersive X‐ray spectrometry (EDS), and corresponding elemental mappings of the (Fe, Ni)OOH‐NF were obtained using a scanning electron microscope (SEM) (Magellan400, FEI) at an accelerating voltage of 10 kV. Transmission electron microscopy (TEM), high‐resolution TEM (HR‐TEM), scanning TEM (STEM), EDS, and fast Fourier transformation (FFT) patterns were acquired with a field emission TEM (Talos F200X, FEI) at 200 kV. The amorphous and crystalline phase structures were analyzed by X‐ray diffractometry (XRD) (SmartLab, RIGAKU) with a 45 kV, 200 mA source in transmission mode over a 2θ range of 20°–80° at a scan speed of 5°/min. X‐ray photoelectron spectroscopy (XPS) (Axis‐Supra, Kratos) determined the chemical compositions and valence states, with profiles calibrated using the C 1s signal. In situ Raman spectra were recorded with a Raman/PL System (LabRAM HR Evolution Visible_NIR, HORIBA) using a 532 nm wavelength light source at room temperature, using a Pt wire and Hg/HgO electrode as counter and reference electrodes, respectively, in a 99.99% 0.1 m KOH electrolyte. Dynamic light scattering (DLS) measurements were performed using a Malvern Instruments ZEN3600 at 25°C with a scattering angle of 90°. Samples were prepared by dispersing the Fe‐containing material in deionized water to obtain a concentration of 0.1 wt.% (Fe basis). The dispersions were sonicated for 1 h to ensure homogeneous suspension and then transferred to standard disposable polystyrene cuvettes for measurement. The concentrations of Fe and Ni in the electrolytes and cell components were quantified by inductively coupled plasma mass spectrometry (ICP‐MS, iCAP RQ, Thermo Fisher Scientific). Electrolyte samples were collected from the anode and cathode compartments before and after the voltage‐cycling activation and durability tests and were subsequently diluted with 2 wt.% HNO_3_ prepared from trace‐metal grade concentrated HNO_3_ and deionized water.

## Conflicts of Interest

The authors declare no conflicts of interest.

## Supporting information




**Supporting File**: advs76261‐sup‐0001‐SuppMat.pdf.

## Data Availability

The data that support the findings of this study are available from the corresponding author upon reasonable request.
