# Supplementary material for: Operando Electrochemical Formation of Integrated Ni‐Fe Oxyhydroxide Anode for Durable Anion Exchange Membrane Water Electrolyzer
Source: Adv Sci (Weinh). 2026 Jun 22:e00055. Online ahead of print. doi: 10.1002/advs.202600055 (PMC13336819; doi:10.1002/advs.202600055)
Supplement: Supplementary file 1 — Supporting File: advs76261‐sup‐0001‐SuppMat.pdf. [file ADVS-9999-e00055-s001.pdf]

Supporting Information

Operando Electrochemical Formation of Integrated Ni-Fe Oxyhydroxide Anode for Durable Anion Exchange Membrane Water Electrolyzer

Euntaek Oh[a], Jonghyun Hyun[a], Hojin Lee[a,e], Changsoo Lee[c], Jang Yong Lee[d], Dong Wook Lee[a], Kyunghwa Seok[a], Jeesoo Park[a], Susung Kim[a], Gisu Doo[b] and Hee-Tak Kim[a*]*

*Corresponding authors: Email: heetak.kim@kaist.ac.kr; Tel.: +82-42-350-3916; Fax: +82-42-350-3910

*Corresponding authors: Email: dooanything@kier.re.kr; Tel.: +82-42-350-3351

[a]Department of Chemical and Biomolecular Engineering, Korea Advanced Institute of Science and Technology (KAIST), Daejeon 34141, Republic of Korea. E-mail: heetak.kim@kaist.ac.kr

[b]Hydrogen Research Department, Korea Institute of Energy Research (KIER), 152 Gajeong-ro, Yuseong-gu, Daejeon 34129, Republic of Korea

[c] Department of Chemical Engineering Education, Chungnam National University, 99 Daehak-ro, Daejeon 34134, Republic of Korea

[d]Department of Chemical Engineering, Konkuk University, Seoul, 05029 Republic of Korea

[e]Clean Energy Research Center, Korea Institute of Science and Technology (KIST), Hwarang-road 14-gil 5, Seongbuk-gu, Seoul 02792, Republic of Korea

Supplementary Note 1. iR compensation

The measured polarization curves were analyzed by separating the cell voltage into kinetic, ohmic, and mass-transport contributions. The ohmic loss was quantified using the high-frequency resistance (HFR) obtained from GEIS Nyquist spectra at each current density. The corresponding iR drop was calculated via Ohm's law (Eq. S1) and used to generate the iR-corrected polarization curves.

$$ohmic\ overpotential = i\ (A\ cm^{-2}) \times HFR\ (Ohm\ cm^{2}) \qquad (Eq. S1)$$

The kinetic overpotential was estimated from Tafel behavior (Eq. S2, S3).

$$\eta_{kin} = \frac{RT}{\alpha nF} \ln\left(\frac{i}{i_0}\right) \qquad (Eq. S2)$$

$$\eta_{kin} = a + b\log(i) \qquad (Eq. S3)$$

For linear fitting, the two lowest-current data points were excluded, and five data points in the 20–90 mA cm^{-2} range were used, yielding a fit quality of $R^2 > 0.99$. For each current density, the kinetic overpotential was calculated by subtracting the reversible potential (1.20 V at 60 °C) from the potential obtained from the Tafel line. The remaining overpotential was assigned to the mass-transport overpotential.

Supplementary Note 2. Crossover of Fe species

Potential Fe crossover and its possible influence on the membrane and cathode during operando Fe-assisted activation were examined using time-resolved electrolyte analysis and post-activation characterizations. The Fe-containing electrolyte was applied exclusively during the voltage-cycling activation (1.54–1.76 V, 50 mV s^{-1}, 800 cycles; ~2 h). ICP-MS analysis of the cathode effluent showed that the Fe concentration in the cathode effluent remained at the impurity level (13–18 μg L^{-1}) throughout the activation, with no increasing trend, which is far lower than the Fe concentration in the anolyte (~ 10^5 μg L^{-1}) (Figure S6a). Consistent with this negligible crossover signature, XPS analysis of the PtRu/C cathode after activation showed no detectable Fe 2p signals (Figure S6b), and SEM images revealed no discernible morphological changes on the cathode (Figure S7a, b). In agreement with the surface analyses, half-cell HER measurements of PtRu/C electrodes showed no meaningful change in HER activity before and after the activation step (Figure S8), thereby excluding cathode poisoning or performance loss induced by Fe species during the operando activation process.

Supplementary Note 3. Ultrasonication test for interfacial robustness

To further evaluate the interfacial robustness of the operando-fabricated VC-800 NF electrode, an ultrasonication test was performed and compared with a conventional spray-coated particle catalyst electrode. The VC-800 NF electrode was immersed in deionized water and subjected to ultrasonication at 200 W for 4 h. For comparison, a spray-coated NiFe alloy catalyst electrode prepared on Ni foam with a polymeric ionomer binder was treated under the same conditions. The electrodes before and after ultrasonication were characterized by SEM and XPS.

Supplementary Note 4. Faradaic efficiency

The OER Faradaic efficiency was calculated according to:

$$\mathrm{FE}_{O_2}(\%) = \frac{4F\left(P - P_{vap,1M\ KOH}\right)V_{O_2}}{RTIt} \times 100$$

where F is the Faraday constant, P is the ambient pressure, $P_{\mathrm{vap,1M\ KOH}}$ is the vapor pressure of the 1 M KOH solution at the measurement temperature, V_{O_2} is the measured oxygen volume, R is the gas constant, T is the gas temperature, I is the applied current, and t is the electrolysis time. Using a measured O_2 volume of 82 mL at approximately 35 °C under 1 atm during galvanostatic operation at 1 A cm $^{-2}$ for 5 min with a 4 cm^2 cell, the OER Faradaic efficiency was determined to be approximately 99.9 %.

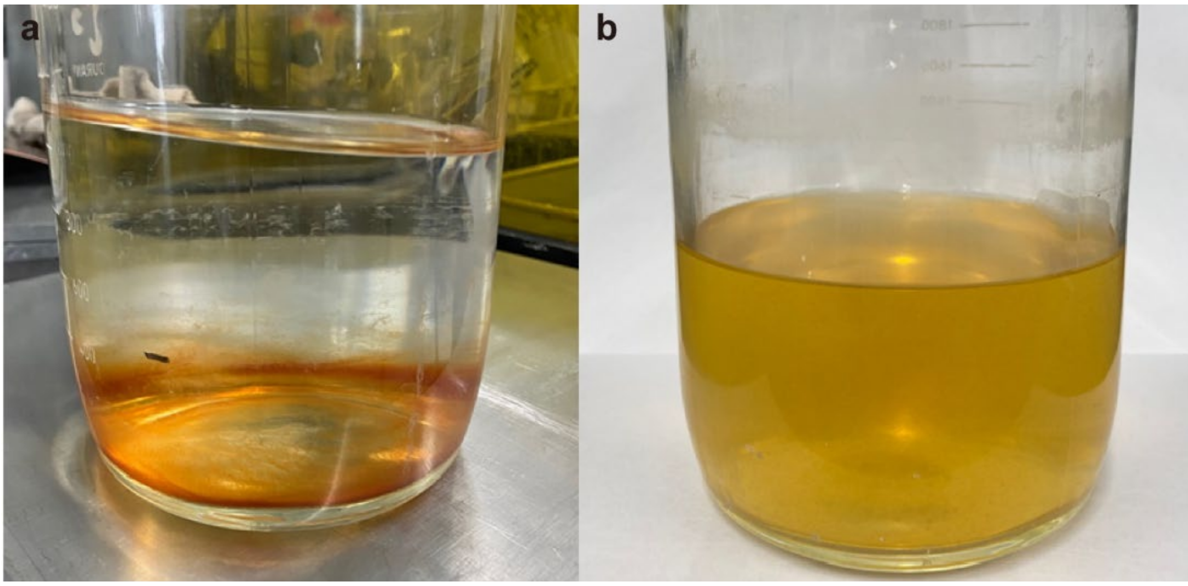

Figure S1. Optical images of 10 ppm $FeCl_2$ + KOH solution. (a) Immediately after the addition of $FeCl_2$. (b) After physical agitation and mixing of the solution.

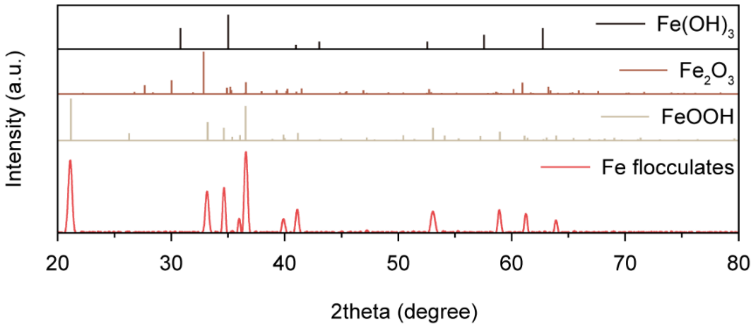

Figure S2. XRD patterns of bulk Fe flocculates, showing peaks corresponding to FeOOH (JCPDS card no. 01-076-7163), Fe_2O_3 (JCPDS card no. 01-086-5600), and $Fe(OH)_3$ (JCPDS card no. 22-0346)

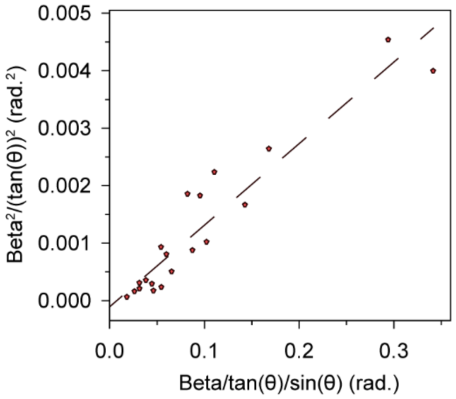

Figure S3. Corresponding W–H plot (βcosθ vs 4sinθ) with a linear fit (equation shown), where the intercept represents size-related broadening and the slope reflects microstrain. (Inset: Summary of the crystallite size and microstrain extracted from Williamson–Hall (W–H) analysis for the goethite phase).

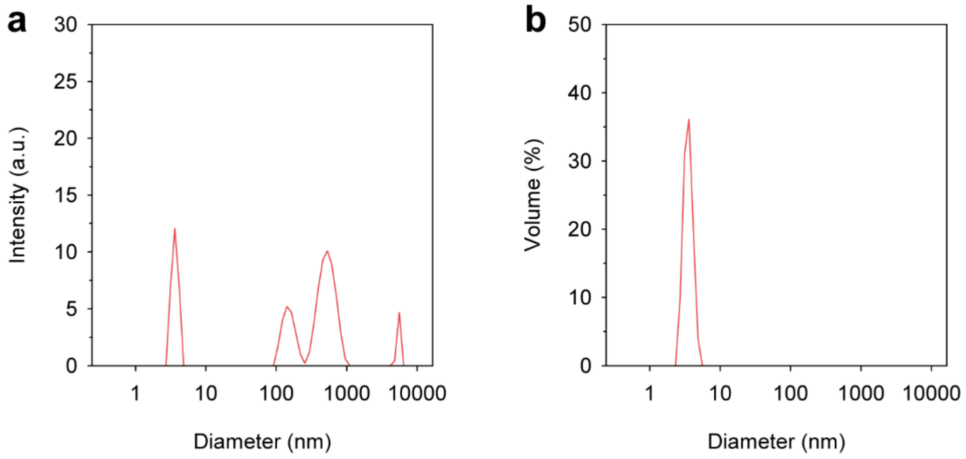

Figure S4. Dynamic light scattering (DLS) analysis of FeOOH species in an Fe-containing electrolyte dispersed in water. Particle-size distributions are presented as (a) intensity-weighted and (b) volume-weighted distributions.

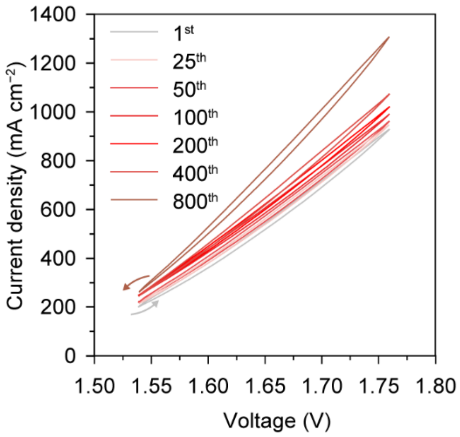

Figure S5. CV curves (50 mV s^{-1}, 1.54–1.76 V) during the electrochemical activation of the anodes in the single cell using 1 M Fe-containing KOH. Solid arrows represent the sweeping direction.

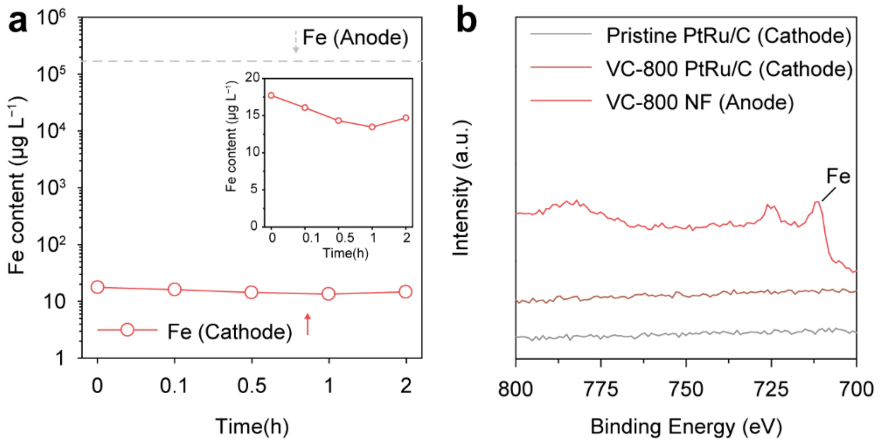

Figure S6. (a) ICP-MS analysis of Fe concentrations in the cathode effluent comparing with that in the anolyte (dot line) during the operando voltage-cycling activation. (b) XPS Fe 2p spectra for the VC-800 NF anode and the PtRu/C cathodes before and after the operando activation.

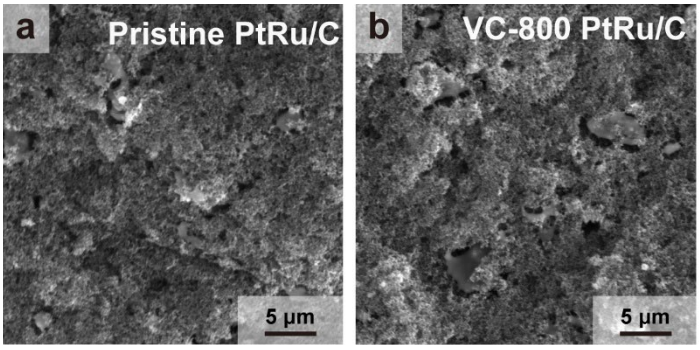

Figure S7. SEM images of PtRu/C cathodes (a) before and (b) after the operando activation.

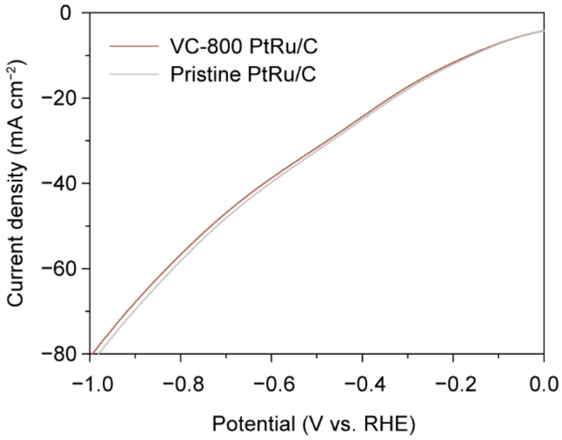

Figure S8. HER performance comparison of the pristine and operated PtRu/C cathodes in a half-cell configuration. The polarizations were measured in 1 M KOH and at room temperature.

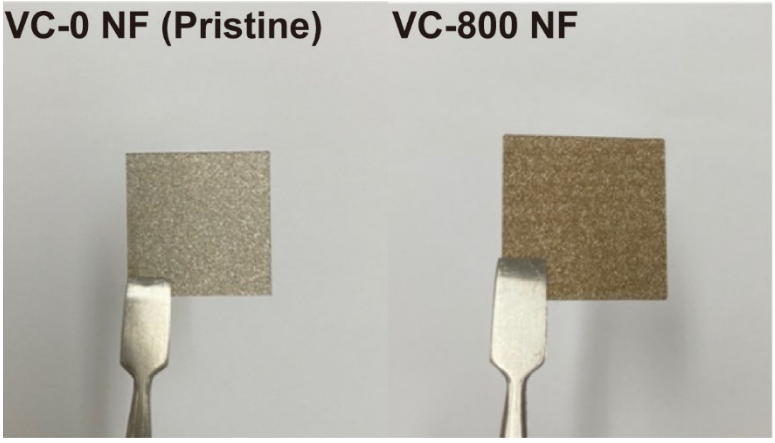

Figure S9. Optical images of the Ni foam. The VC-0 NF is shown on the left, and the VC-800 NF is displayed on the right.

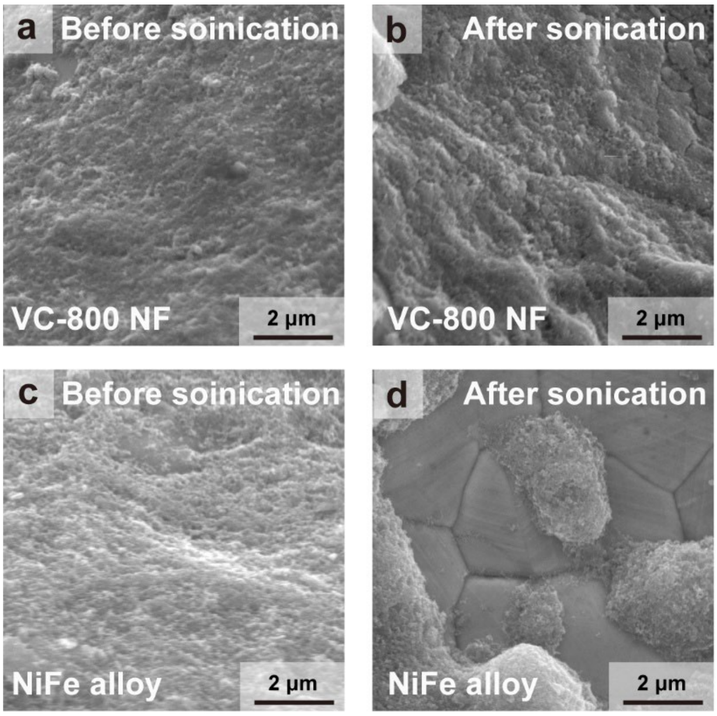

Figure S10. SEM images of VC-800 NF (a) before and (b) after ultrasonication; NiFe alloy particles sprayed on nickel foam (c) before and (d) after ultrasonication.

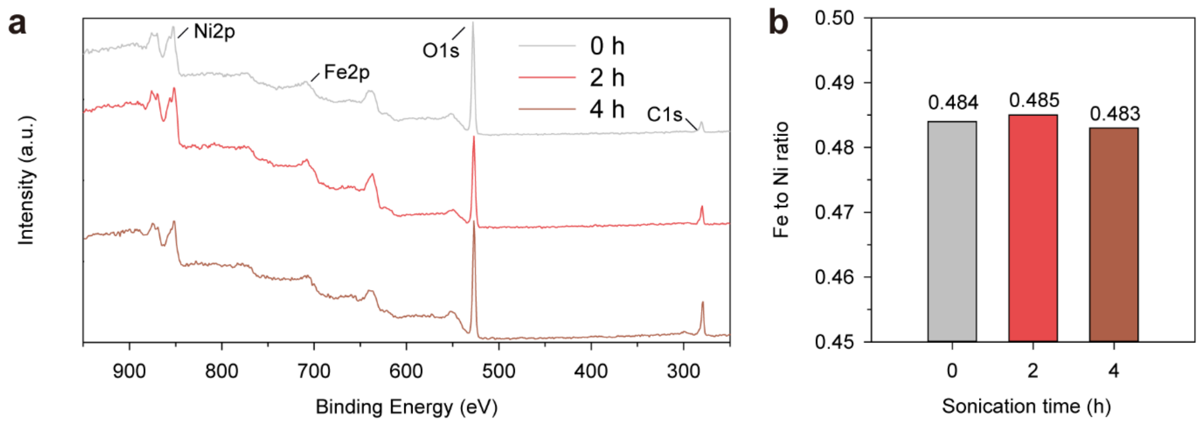

Figure S11. (a) XPS survey spectra of the VC-800 NF collected during the 2 hours of ultrasonication. (b) Fe to Ni atomic ratios of VC-800 NF as a function of ultrasonication time.

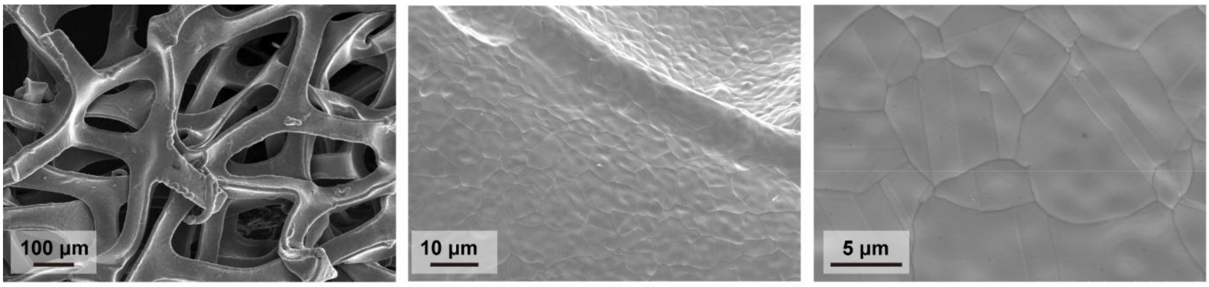

Figure S12. SEM images of the surface of pristine nickel foam (VC-0 NF) at different magnifications.

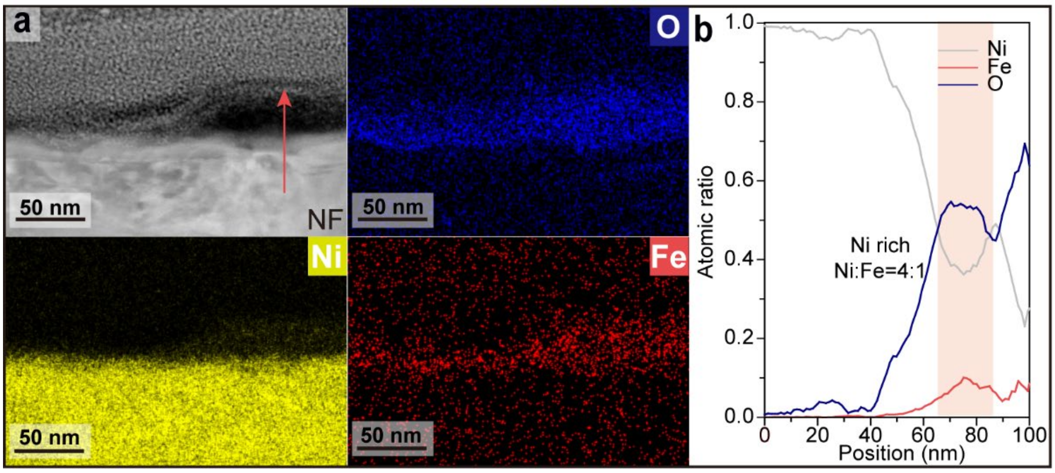

Figure S13. (a) STEM image and EDS elemental mapping for VC-50. (b) Atomic distribution across the VC-50 NF cross-section obtained from EDS line-scan analysis.

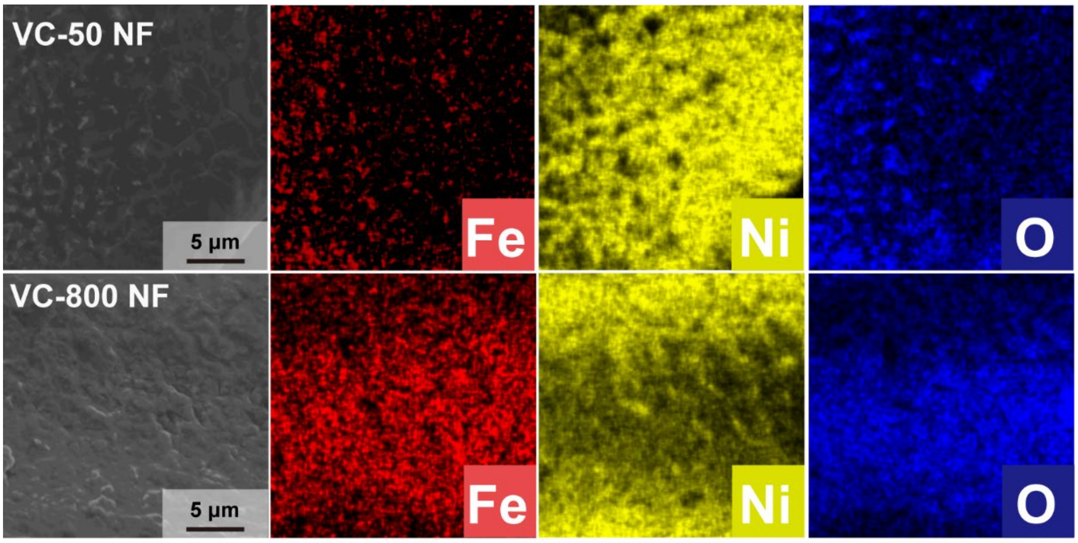

Figure S14. SEM and EDS images of the VC-50 and VC-800 NF electrode surfaces.

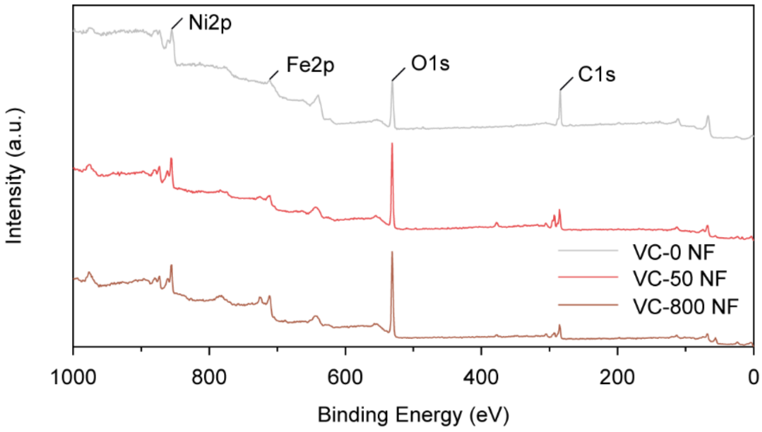

Figure S15. XPS survey spectra of Ni 2p, Fe 2p, O 1s, and C 1s for the VC- 0 NF, VC-50 NF and VC-800 NF.

	Name	Peak (BE)	FWHM (eV)	Area (CPS*eV)	Atomic (%)
**VC-0 NF**	O1s	530.8	3.4	1004386	35.8
	Ni2p	854.6	5.7	1219059	12.0
	C1s	284.2	2.6	582491	50.2
	Fe2p	711.0	5.6	247580	2.1
**VC-50 NF**	O1s	531.0	2.9	1343305	51.8
	Ni2p	855.8	3.6	1060229	11.3
	C1s	285.0	2.7	326027	30.4
	Fe2p	711.4	7.2	709392	6.4
**VC-800 NF**	O1s	530.9	2.9	1418357	55.9
	Ni2p	855.5	2.1	984559	10.7
	C1s	285.0	2.9	230962	22.0
	Fe2p	711.4	5.6	1224609	11.3

Table S1. Information of full width at half maximum (FWHM), area, and atomic ratio calculated from XPS survey spectra for the VC- 0 NF, VC-50 NF, and VC-800 NF

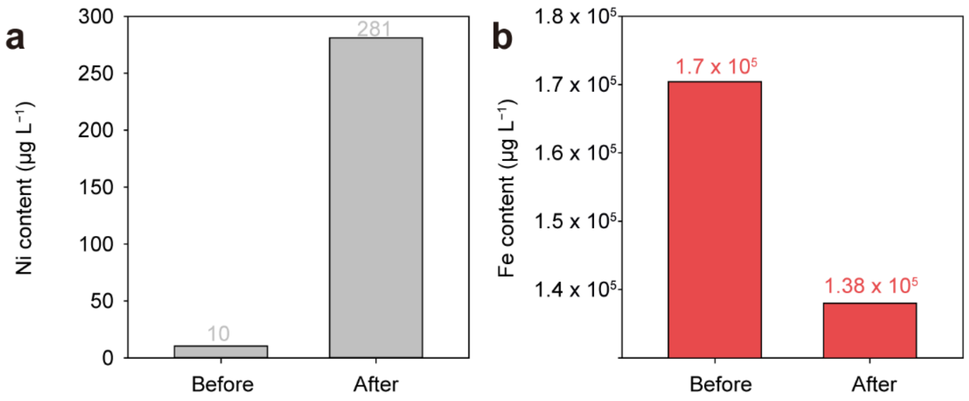

Figure S16. Inductively coupled plasma-mass spectrometry (ICP-MS) analysis of the electrolyte used for synthesizing (Fe, Ni)OOH NF. The data show the changes in Ni and Fe concentrations before and after 800 cycles of voltage-cycling.

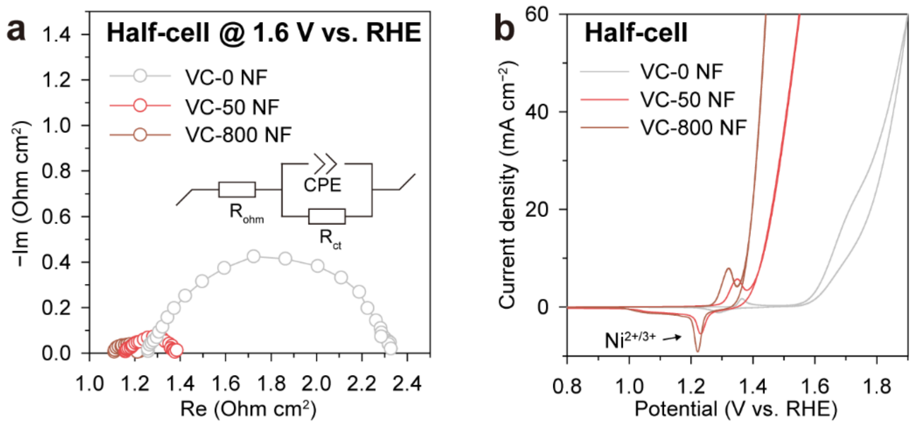

Figure S17. (a) Nyquist plots at 1.6 V vs. RHE over a frequency range of 100 kHz to 100 mHz for VC-0 NF, VC-50 NF, and VC-800 NF in 1 M KOH in a half-cell (Inset: equivalent circuit model used for quantification of R_{ct} and R_{ohm}). (b) CV curves at a scan rate of 50 mV s^{-1} for VC-0 NF, VC-50 NF, and VC-800 NF.

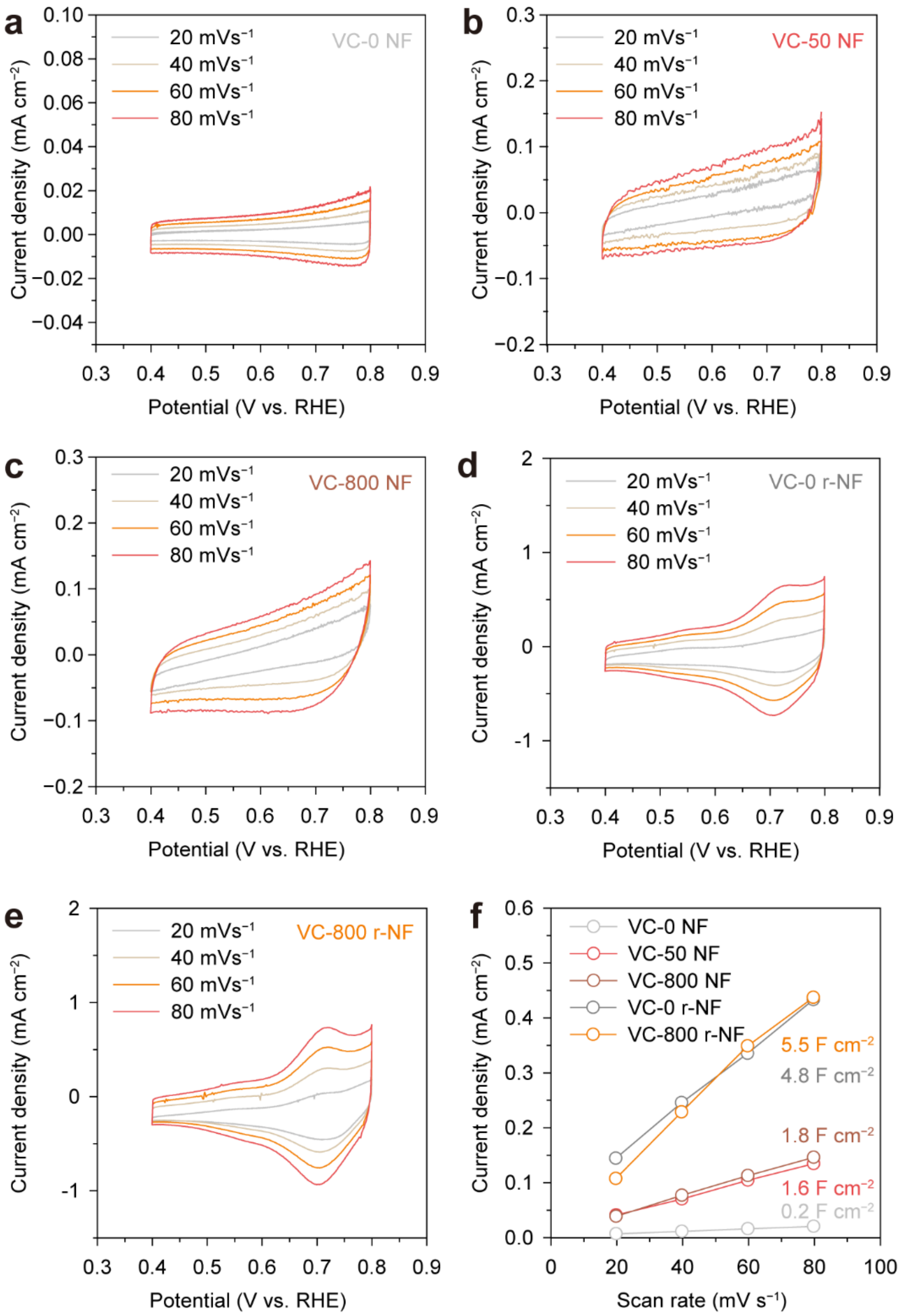

Figure S18. CV curves measured in the non-faradaic potential region at scan rates ranging from 20 to 80 mV s^{-1} for (a) VC-0 NF, (b) VC-50 NF, (c) VC-800 NF, (d) VC-0 r-NF, and (e) VC-800 r-NF electrodes. (f) Linear dependence of the capacitive current density on scan rate, extracted from data in (a-e). The corresponding electrochemical double-layer capacitance (C_{dl}) values are indicated.

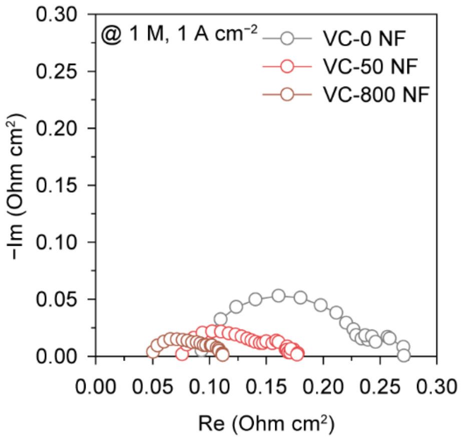

Figure S19. Nyquist plots of the impedance at 1 A cm^{-2} over a frequency range of 100 kHz to 100 mHz for VC-0 NF, VC-50 NF, and VC-800 NF in 1 M KOH in a single-cell

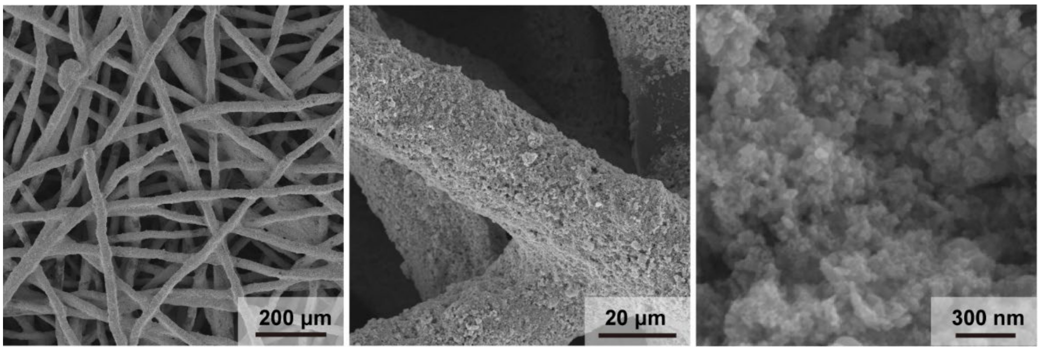

Figure S20. SEM surface image of the IrO_x-based catalyst layer with 10 wt. % QPC-TMA ionomer.

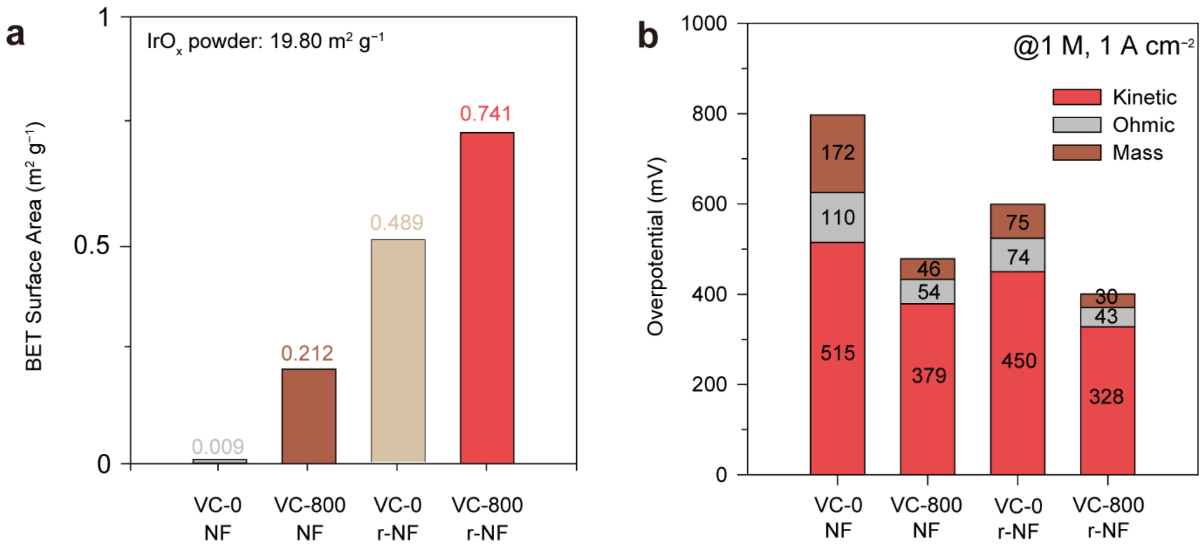

Figure S21. (a) Specific surface area measurements for VC-0 NF, VC-800 NF, VC-0 r-NF, and VC-800 r-NF, analyzed using the Brunauer–Emmett–Teller (BET) method. (b) Deconvolution of the overpotential at 1 A cm^{-2} from the polarization curves shown in Figure 5c and 5e.

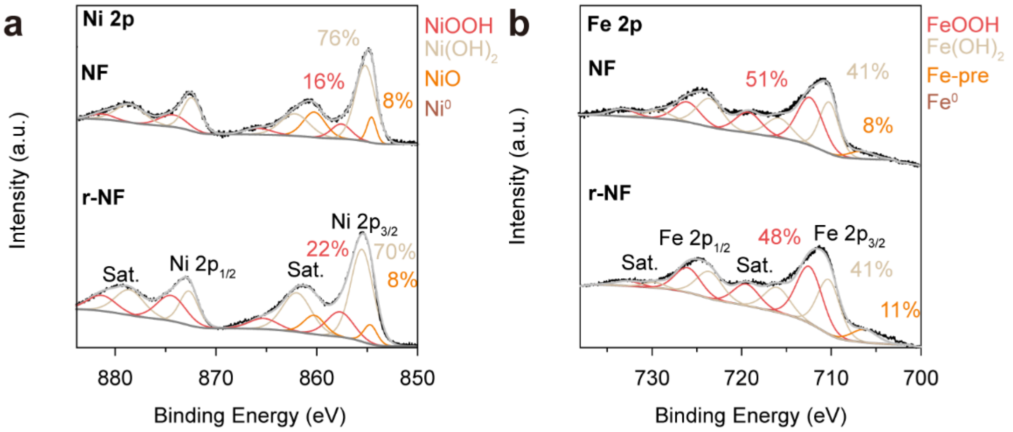

Figure S22. Chemical-state analysis of the integrated VC-800 NF and r-NF. (a–b) High-resolution XPS spectra and peak deconvolution for (a) Ni 2p and (b) Fe 2p collected from the VC-800 NF and VC-800 r-NF anode.

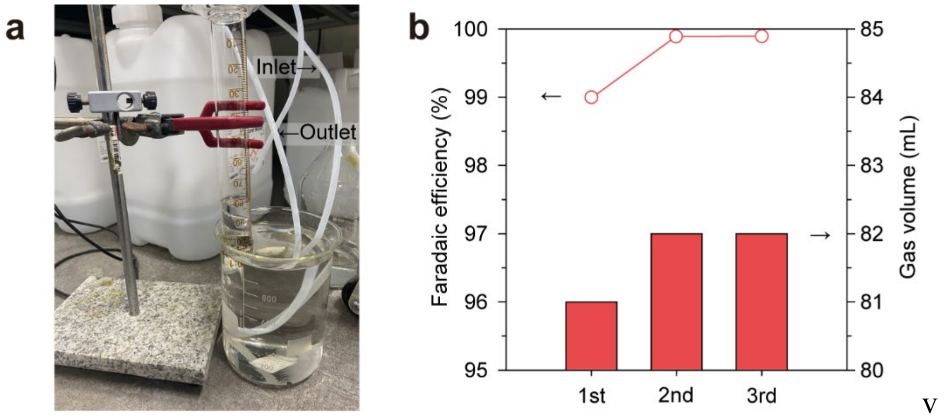

Figure S23. (a) Photograph of the O_2 collection setup used for Faradaic efficiency measurement of the VC-800 r-NF electrode. (b) Faradaic efficiency of the VC-800 r-NF measured by volumetric O_2 collection, with the results from the first, second, and third trials indicated.

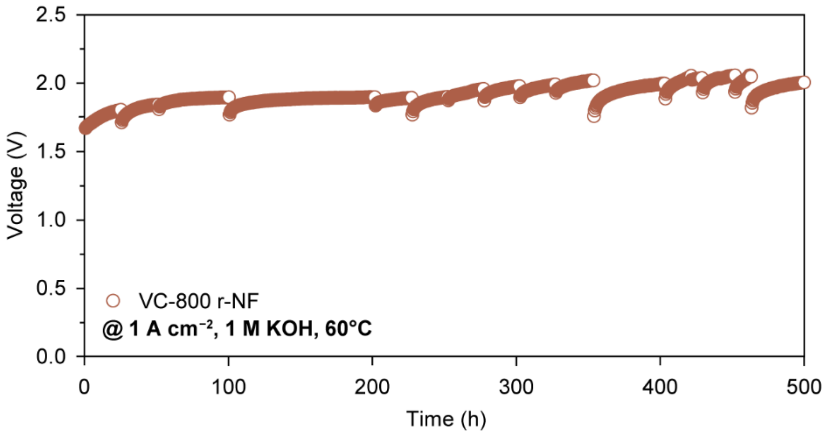

Figure S24. Long-term stability test of the (Fe, Ni)OOH NF single-cells. Chrono-potentiometry (CP) data of VC-800 r-NF measured at a constant current density of 1 A cm^{-2} in 1 M KOH at 60 °C.

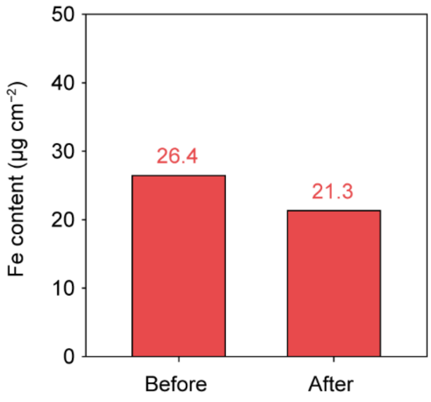

Figure S25. Fe content in the VC-800 NF electrodes before and after the durability test of 300 h using the ICP-MS analysis.

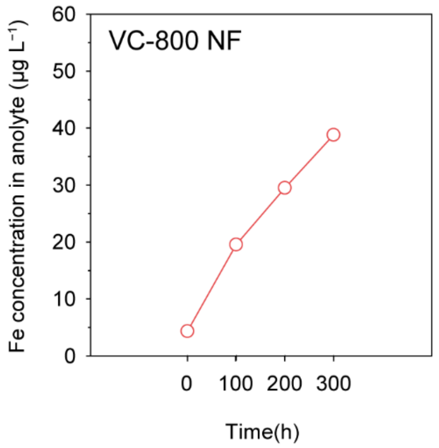

Figure S26. Fe concentrations in the anolyte during the durability test. The Fe precursor was not intentionally added to the electrolyte after the electrode activation step.

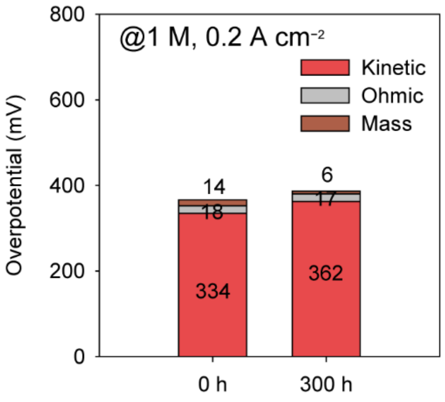

Figure S27. Deconvolution of the overpotential at 0.2 A cm^{-2} from the polarization curves of VC-800 NF after 0 h and 300 h, shown in Figure 6c.

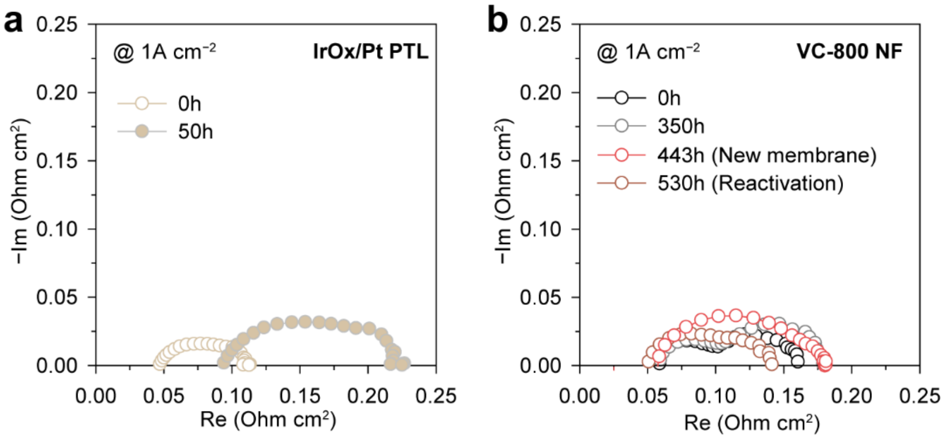

Figure S28. Nyquist plots measured at 1 A cm^{-2} for (a) IrO_x/Pt PTL and (b) VC-800 NF during the durability test in 1 M KOH at 60 °C.

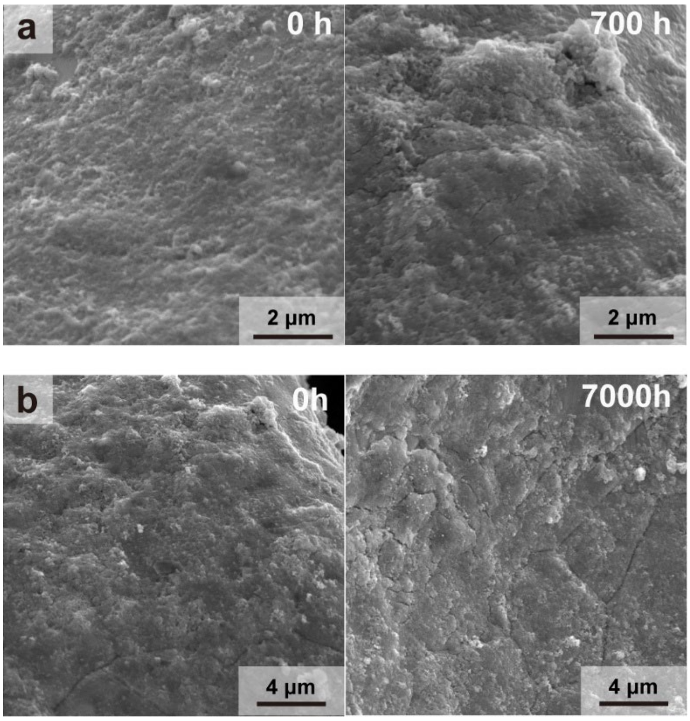

Figure S29. SEM images of the VC-800 NF before and after the durability test. (a) Morphology of the electrode operated in a single cell under 1 M KOH at 1 A cm^{-2} for 700 h and (b) operated in a stack cell under 0.1 M KOH at 0.5 A cm^{-2} before and after 7000 h.

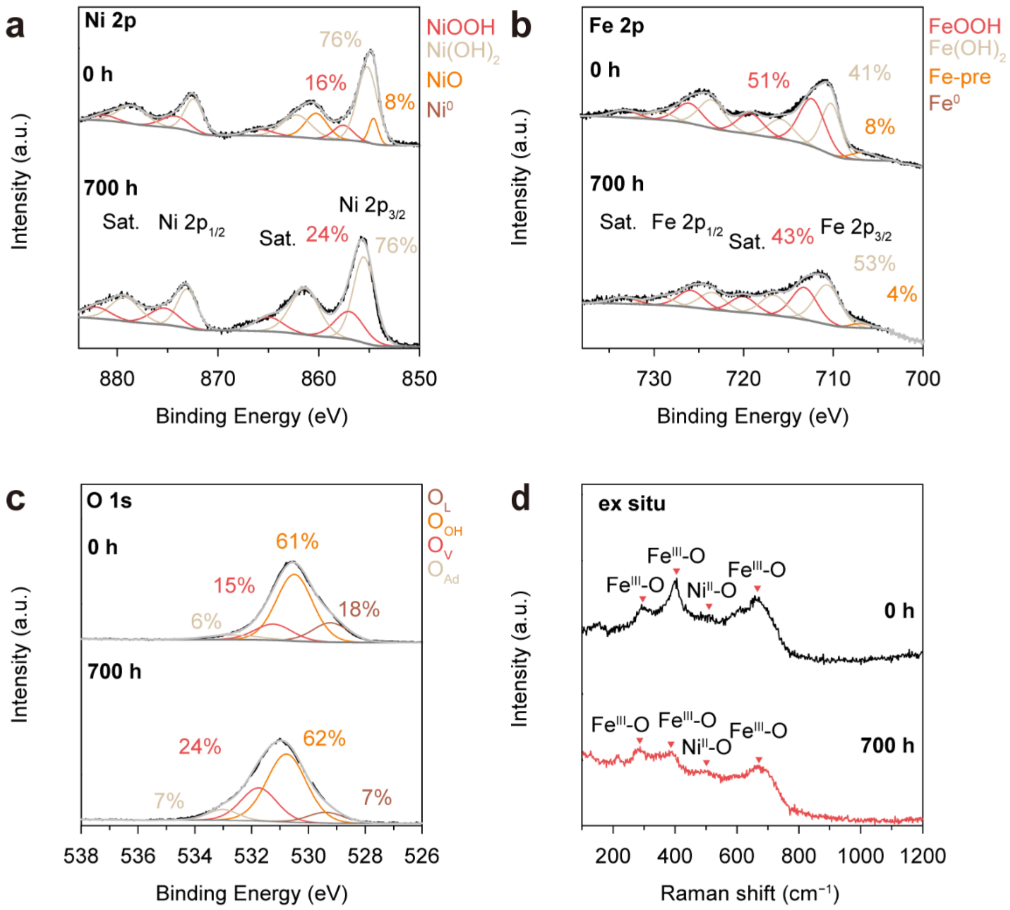

Figure S30. Post-durability chemical-state analysis of the integrated VC-800 NF operated in a single cell. (a–c) High-resolution XPS spectra and peak deconvolution for (a) Ni 2p, (b) Fe 2p, and (c) O 1s collected from the VC-800 NF anode before (0 h) and after (700 h) durability test. (d) Ex situ Raman spectra of the same electrodes at 0 h and 700 h.

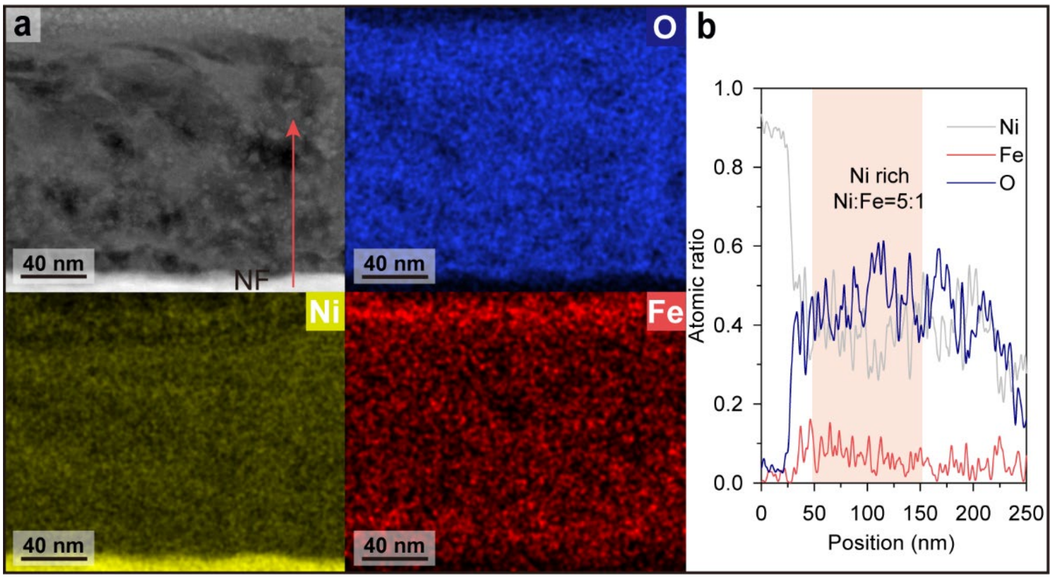

Figure S31. (a) STEM image and EDS elemental mapping for VC-800 NF after durability test for 700 h. (b) Atomic distribution across the VC-800 NF cross-section obtained from EDS line-scan analysis.

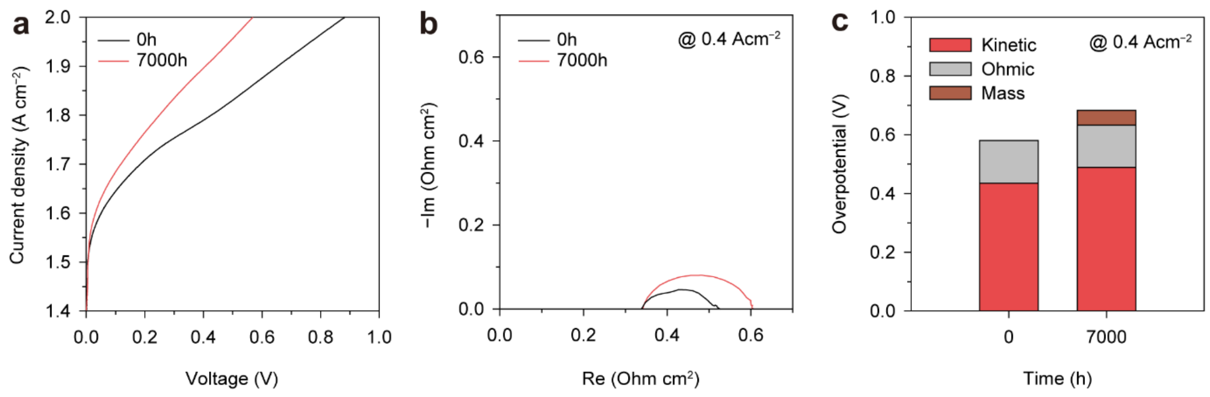

Figure S32. Electrochemical characterization of the VC-800 NF cell before and after the 7000 h durability test. (a) Linear sweep voltammetry curves, (b) Nyquist plots at 0.4 A cm^{-2}, and (c) overpotential breakdown at 0.4 A cm^{-2}.

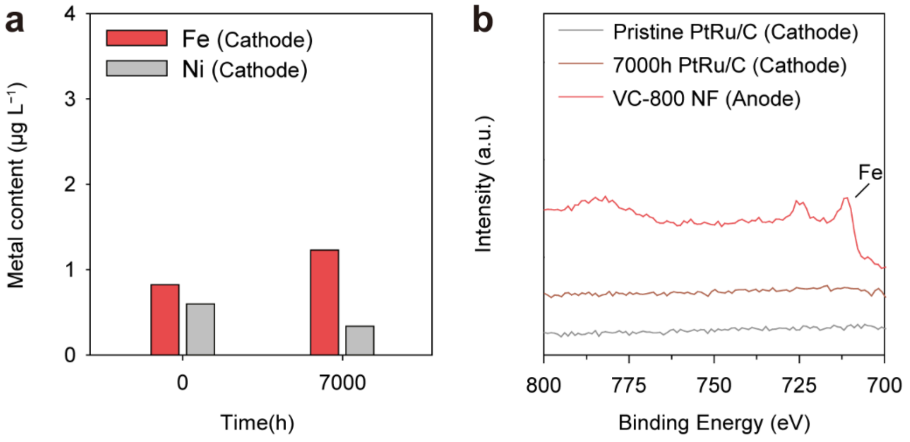

Figure S33. (a) ICP–MS analysis of Fe and Ni concentrations in the cathode effluent before and after 7000 h of operation. (b) XPS Fe 2p spectra for the cathode surfaces before and after 7000 h of operation and the VC-800 NF anode surface after operando activation.

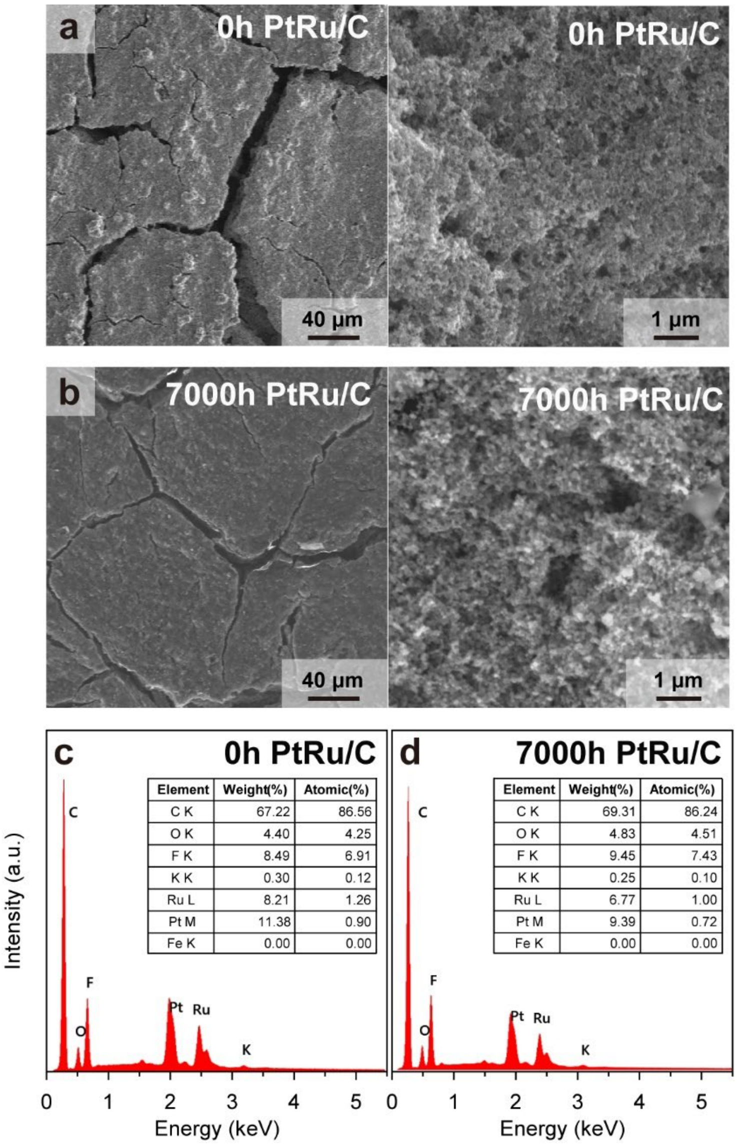

Figure S34. SEM images of the PtRu/C cathode surface used in the two-cell stack operation. (a) Pristine (0 h) and (b) after 7000 h of durability test. (c, d) Corresponding EDS spectra with the quantitative elemental compositions as the inset.

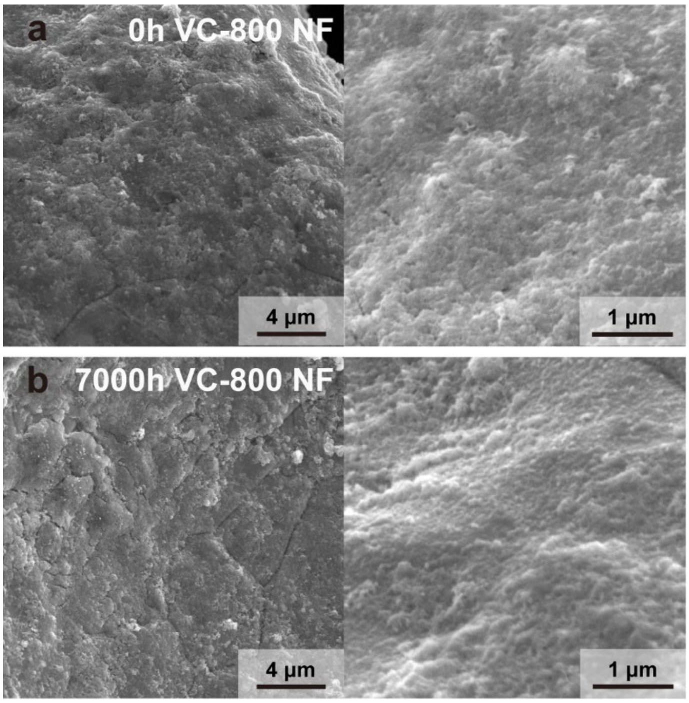

Figure S35. Surface SEM images of the VC-800 NF anodes (a) before and (b) after the 7000 h durability test.

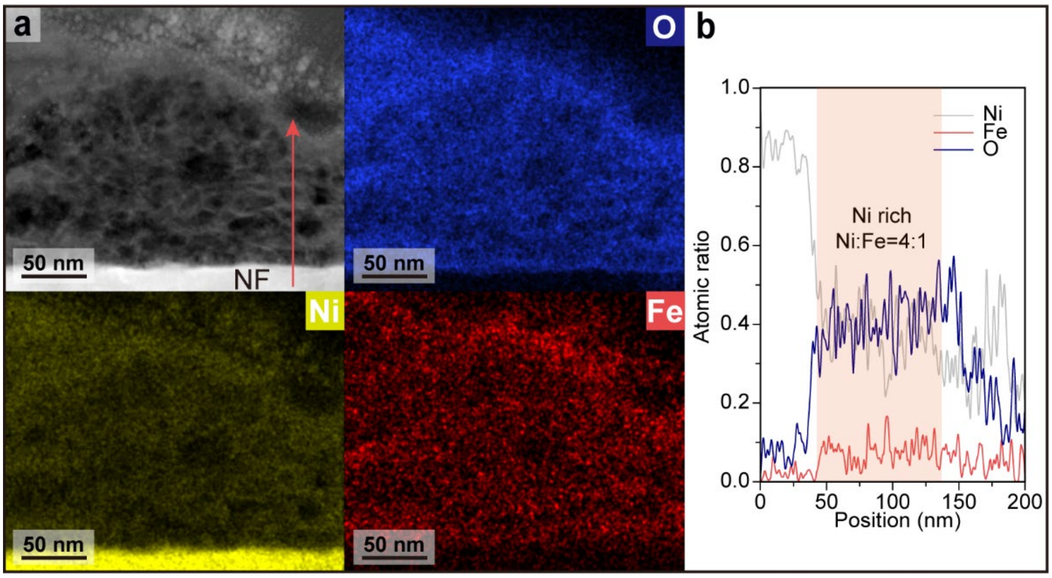

Figure S36. (a) STEM image and EDS elemental mapping for VC-800 NF after the 7000 h durability test. (b) Atomic distribution across the VC-800 NF cross-section obtained from EDS line-scan analysis.

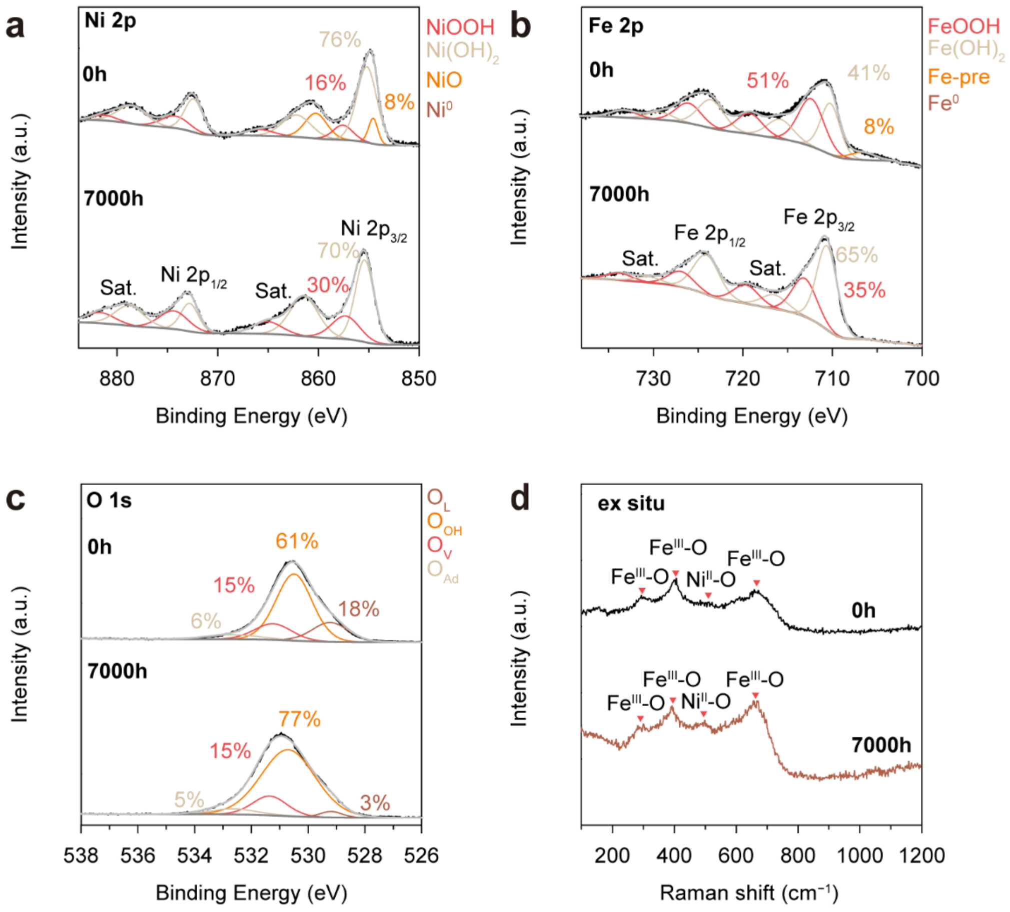

Figure S37. Post-durability chemical-state analysis of the integrated VC-800 NF. (a–c) High-resolution XPS spectra and peak deconvolution for (a) Ni 2p, (b) Fe 2p, and (c) O 1s collected from the VC-800 NF anode before (0 h) and after the 7000 h durability test. (d) Ex situ Raman spectra of the same electrodes at 0 h and 7000 h.

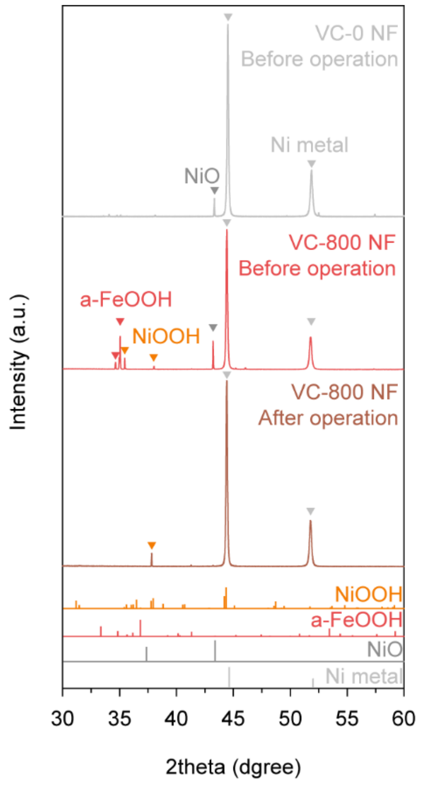

Figure S38. XRD of the VC-0 NF, VC-800 NF 0 h (before durability test), and VC-800 NF after durability test for 7000 h.

NiFe based catalyst loading (mg cm^{-2})	Membrane	Current density at 1.8 V (A cm^{-2})	Durability test			KOH Conc., Temp.	Active area (cm^{2})	Method	Ref
			Applied current density (mA cm^{-2})	Voltage decay (mV kh^{-1})	Time (h)				
0.25	QPC-TMA (30-80 µm)	2.8	625	39	7000	1 M, 60 °C	4	Electro-deposition (binder-free)	This work
0.5	FAA-3-50 (50 µm)	1.4–1.6	100	800–900	200	1 M, 70 °C	5	Electro-deposition (binder-free)	[13]
0.5	FAA-3-50 (50 µm)	2.4–2.6	100	5500–6000	70	1 M, 70 °C	5	Spray-coated catalyst particles (binder)	[13]
4.8	PAP (20 µm)	1	500	1810	70	1 M, 80 °C	6.25	Spray-coated catalyst particles (binder)	[40]
2	DURAIO-N (70 µm)	1–1.2	1000	84	1000	1 M, 60 °C	5	Spray-coated catalyst particles (binder)	[41]
20	PFTP-13 (30 µm)	0.9	500	560	1000	1 M, 80 °C	6.25	Electro-deposition (binder)	[42]
0.25	b-PDTP-Trip-5 (20-50 µm)	3.2–3.3	1500	50	2000	1 M, 60 °C	5	Spray-coated catalyst particles (binder)	[43]
0.25	PDTP (20 µm)	3.9–4.1	1000	170	870–900	1 M, 80 °C	5	Spray-coated catalyst particles (binder)	[44]
2	AF3-HWK9-	1.2–1.3	1000	800	100	1 M, 70 °C	5	Spray-coated catalyst	[45]

	75 (75 μm)							particles (binder)	
2	FAA-3-50 (50 μm)	1.4–1.5	1000	250–300	600	1 M, 60 °C	25	Spray-coated catalyst particles (binder)	[46]
5	AF1-HNN8-50 (50 μm)	1.1–1.2	1000	120	550	1 M, 60 °C	4–5	Spray-coated catalyst particles (binder)	[47]

Table S2. Comparisons of the AEMWE performance and durability, along with the cell configuration in the literature.

Catalyst	Substrates and reagents	Quantity used (cm^2 or mg or mmol)	Unit price (US $ per cm^2 or g or mL)	Cost (US $)	Total cost (US $ per cm^2)	Method
(Fe, Ni)OOH NF	Nickel foam	4 cm^2	0.00568	0.02270	0.0057	This work
	$FeCl_2·4H_2O$	2 mg	0.04053	0.00008		
$Fe_{11\%}$-NiO/NF [54]	Nickel foam	4 cm^2	0.00568	0.02270	1.0480	Hydrothermal deposition at 120 °C for 8 h; CVD at 350 °C for 2 h
	$Ni(NO_3)_2·6H_2O$	3 mmol	4.77600	4.16600		
	$FeCl_3·6H_2O$	0.4 mmol	0.03210	0.00347		
Cu@NiFe LDH/CF [55]	Copper foam	10 cm^2	0.01340	0.13400	2.2350	CVD at 180 °C for 1 h; Electrochemical reduction at -0.4 V; Electrodeposition at -1.0 V for 90 s
	$(NH_4)_2S_2O_8$	10 mmol	0.56900	1.29800		
	$Ni(NO_3)_2·6H_2O$	15 mmol	4.77600	20.83200		
	$FeSO_4·7H_2O$	15 mmol	0.02140	0.08920		
NiFeOx/IF [56]	Iron foam	2.5 cm^2	0.01890	0.04730	0.0729	CV-Treated at 1.1-1.4 V; phosphorization
	$NiSO_4·6H_2O$	1 mmol	0.51500	0.13500		
NiFe-OH/NF [9]	Nickel foam	6 cm^2	0.00568	0.03410	0.0165	Redox and hydrolysis co-precipitation for 10 h
	$Fe(NO_3)_3·9H_2O$	5 mmol	0.03210	0.06480		

Table S3. Comparison of the cost and fabrication methods of previously reported high-performance self-supported NiFe-based OER electrocatalysts.

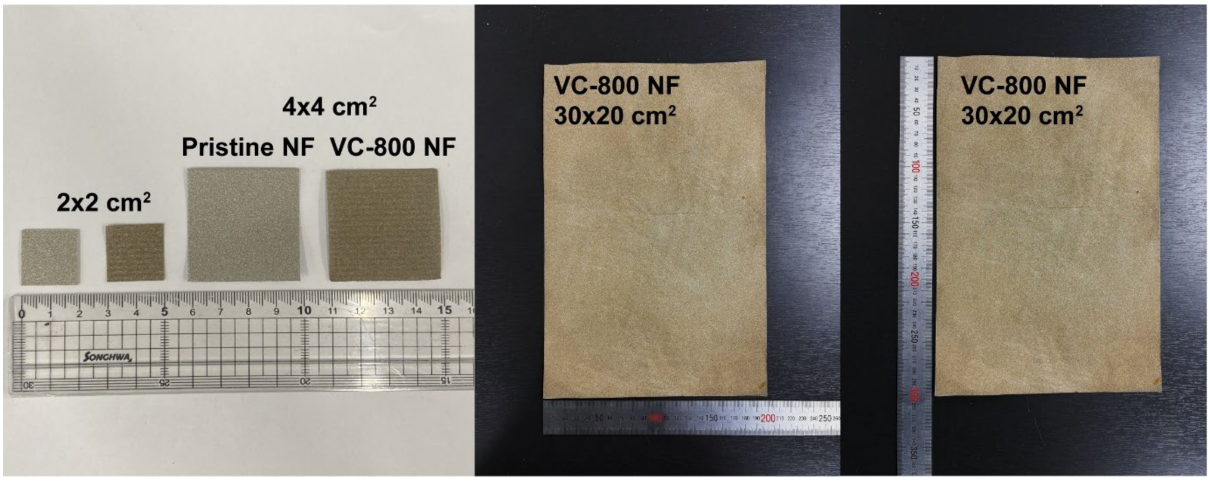

Figure S39. Optical images of VC-800 NF and pristine NF were fabricated at different scales.
